# The Tumor Suppressor DAB2IP Is Regulated by Cell Contact and Contributes to YAP/TAZ Inhibition in Confluent Cells

**DOI:** 10.3390/cancers15133379

**Published:** 2023-06-27

**Authors:** Mattia Apollonio, Arianna Bellazzo, Nicoletta Franco, Silvia Lombardi, Beatrice Senigagliesi, Loredana Casalis, Pietro Parisse, Agnes Thalhammer, Gabriele Baj, Rossella De Florian Fania, Giannino Del Sal, Licio Collavin

**Affiliations:** 1 Department of Life Sciences, University of Trieste, Via L. Giorgieri 1, 34127 Trieste, Italy; 2 Elettra-Sincrotrone Trieste, Area Science Park Basovizza, 34149 Trieste, Italy; 3 Institute of Materials (IOM), Italian National Research Council (CNR), Area Science Park Basovizza, 34149 Trieste, Italy; 4 ICGEB—Area Science Park Padriciano, 34149 Trieste, Italy; 5 Fondazione Istituto FIRC di Oncologia Molecolare (IFOM), 20139 Milan, Italy

**Keywords:** AIP1, contact inhibition, mechanotransduction, cell stiffness, cell-to-cell contact, Hippo pathway, Ras-GAP

## Abstract

**Simple Summary:**

External and internal mechanical forces affect the shape, movement, proliferation and metabolism of cells within tissues. The transcriptional regulators YAP and TAZ are key sensors of mechanical inputs, and their aberrant activation in solid tumors promotes cancer aggressiveness. It is thus important to understand how YAP/TAZ activity is controlled, and how such control is altered in tumors. DAB2IP is a cytoplasmic protein that negatively modulates several signaling pathways; being a tumor suppressor, it is frequently disabled in cancer. Using mammary epithelial cells as a model, we find that DAB2IP levels depend on cell density, and depletion of DAB2IP modifies cell morphology and stiffness in confluent cultures. We also find that DAB2IP behaves as an inhibitor of YAP/TAZ nuclear localization and activity in confluent cells. These observations suggest that DAB2IP may function as a sensor of cellular interactions, contributing to restraining the activation of oncogenic signaling pathways in intact tissues.

**Abstract:**

External and internal mechanical forces modulate cell morphology, movement, proliferation and metabolism, and represent crucial inputs for tissue homeostasis. The transcriptional regulators YAP and TAZ are important effectors of mechanical signaling and are frequently activated in solid tumors, correlating with metastasis, chemoresistance, and shorter patient survival. YAP/TAZ activity is controlled by various pathways that sense cell shape, polarity, contacts, and mechanical tension. In tumors, aberrant YAP/TAZ activation may result from cancer-related alterations of such regulatory networks. The tumor suppressor DAB2IP is a Ras-GAP and scaffold protein that negatively modulates multiple oncogenic pathways and is frequently downregulated or inactivated in solid tumors. Here, we provide evidence that DAB2IP expression is sustained by cell confluency. We also find that DAB2IP depletion in confluent cells alters their morphology, reducing cell packing while increasing cell stiffness. Finally, we find that DAB2IP depletion in confluent cells favors YAP/TAZ nuclear localization and transcriptional activity, while its ectopic expression in subconfluent cells increases YAP/TAZ retention in the cytoplasm. Together, these data suggest that DAB2IP may function as a sensor of cell interactions, contributing to dampening cellular responses to oncogenic inputs in confluent cells and that DAB2IP loss-of-function would facilitate YAP/TAZ activation in intact epithelia, accelerating oncogenic transformation.

## 1. Introduction

Cell communication is crucial for embryonic development, tissue homeostasis, and repair after damage. In addition to soluble molecules such as metabolites, growth factors, and hormones, external and internal mechanical inputs also influence cell behavior and fate [1,2]. In epithelia, cells are connected to each other and the extracellular matrix (ECM) by membrane proteins providing crucial inputs for cell behavior. In particular, focal adhesions (FAs) and adherens junctions (AJs) are essential for tissue homeostasis and cell migration and are dynamically rearranged when the epithelial structure is disrupted, such as in cancer, affecting the cytoskeletal organization and signal transduction [1,3].

YAP (Yes-associated protein 1) and TAZ (WWTR1, WW-domain-containing transcription regulator 1) are two paralogue transcriptional co-activators that control gene expression by interacting with members of the TEAD family of DNA-binding factors [4,5]. YAP/TAZ are regulated by multiple cellular and extracellular signals; once activated, they promote proliferation, survival, and even reprogramming of differentiated cells to a stem-like progenitor condition [6,7,8,9]. In adult tissues, YAP and TAZ are kept in check by multiple negative regulators, including cell junctions and other mechanical inputs. With the onset of non-homeostatic conditions, such as the collapse of physiological cell interactions with the surrounding environment, YAP and TAZ are activated, prompting wound healing and organ regeneration [4,6,7,8,9]. YAP/TAZ are primarily regulated by shuttling between the nucleus and the cytoplasm; in particular, in the cytoplasm, they are held inactive or degraded [4,5]. In cultured cells, such regulation is clearly dependent on mechanical inputs and cell–cell contacts: YAP/TAZ are cytoplasmic when cells are grown on a soft matrix and nuclear when cells are grown on a stiff surface. Additionally, YAP/TAZ are cytoplasmic when cells have a small surface to attach to, but are nuclear when cells can spread on large adhesive areas. Finally, YAP/TAZ are nuclear in low-density cultures, but are retained in the cytoplasm in confluent cells, responding to contact inhibition [10,11]. Aberrant YAP/TAZ activity is pervasive in solid tumors, when cancer cells acquire irregular shapes with altered cytoskeletal dynamics and cell–cell interactions and are often embedded in a fibrotic matrix with increased stiffness. YAP and TAZ have a relevant impact on cancer aggressiveness, as they promote cell cycle progression, epithelial to mesenchymal transition (EMT), cell migration, and chemoresistance [6,7]. Despite many important advancements in the field, some aspects of YAP and TAZ control remain partially undefined, in particular regarding cytoplasmic upstream regulators.

The tumor suppressor DAB2IP (Disabled Homolog 2 Interacting Protein), also called AIP1 (ASK1 Interacting Protein), is a cytoplasmic Ras-GAP and adaptor protein that negatively modulates multiple oncogenic signaling pathways such as Ras-MAPK, PI3K-AKT, NF-kB, and GSK3β-beta catenin, thus affecting the reciprocal interaction between cancer cells and their microenvironment. Accordingly, its loss of function promotes tumor growth, metastasis, and chemoresistance [12,13,14,15]. DAB2IP expression is frequently reduced in tumors by promoter methylation; in addition, post-transcriptional mechanisms of inactivation in cancer and stromal cells have been reported: these include repression by microRNAs, formation of aberrant protein complexes, and possibly increased degradation [12,13,16].

Multiple studies have firmly established that DAB2IP can modulate signaling cascades triggered by growth factors and activated oncogenes [14,17], inflammatory cytokines [18,19], and hormones [20,21]. Intriguingly, recent observations suggest that DAB2IP may also be involved in mechanosensing and cell polarity. First, Raza et al. found that the Drosophila ortholog of DAB2IP, named Raskol, interacts with E-cadherin in epidermal cells of the ovary, and its depletion caused adhesion and migration defects of border cells and polar cells during egg chamber development [22]. Second, Zhang et al. reported that DAB2IP is reduced when colon cancer cells are grown in a soft matrix, so its expression may be affected by ECM stiffness [23]. Prompted by these observations, we wondered whether DAB2IP may respond to mechanical inputs and/or contribute to modulating oncogenic signaling pathways triggered by alterations in tissue structure. Using non-transformed mammary epithelial cells as a model, we found that DAB2IP levels correlate with cell density and cell–cell interactions, and DAB2IP loss-of-function increases YAP activity in confluent cells.

## 2. Materials and Methods

### 2.1. Cell Lines, Culture Conditions, Transfections, Trasductions, and Drug Treatments

*Cell lines and culture conditions.* MCF10A (non-transformed mammary epithelial cells; RRID:CVCL_0598), MDA-MB-231 (triple-negative breast cancer; RRID:CVCL_0062), HMLE (non-transformed mammary epithelial cells) and NIH 3T3 murine fibroblasts (RRID:CVCL_0594) were provided by co-author Giannino Del Sal [24]. RWPE-1 (non-transformed prostate epithelial cells; RRID:CVCL_3791) were kindly provided by Prof. Andrea Lunardi, University of Trento, Trento, Italy [25]. HUVECs (human umbilical vein endothelial cells) were kindly provided by Prof. Roberta Bulla, University of Trieste, Trento, Italy [25]. BJ-5ta (h-TERT immortalized human fibroblasts; CRL-4001), HEK293T (CRL-3216), and Phoenix-GP (CRL-3215) were purchased from ATCC (Manassas, VA, USA).

MCF10A were cultured in DMEM/Ham’s F12 medium (Euroclone, Milano, Italy), supplemented with 5% Horse Serum (ECS0091D, Euroclone), antibiotics (ECB3001D, Euroclone), 10 μg/mL Insulin (Sigma, Burlington, MA, USA), 0.5 μg/mL Hydrocortisone (Sigma), and 20 ng/mL Epidermal Growth Factor (Cell Guidance System, Cambridge, UK). MDA-MB-231, NIH 3T3, HEK293T and Phoenix-GP were cultured in DMEM (Euroclone) supplemented with 10% FBS (ECS0183L, Euroclone), and antibiotics (Euroclone). RWPE-1 were cultured in Keratinocyte-SFM (Gibco, Grand Island, NY, USA) supplemented with 0.05 mg/mL of Bovine Pituitary Extract (Gibco, Grand Island, NY, USA), 5 ng/mL of human recombinant EGF (Gibco) and antibiotics (Euroclone). HUVECs were isolated by collagenase treatment, cultured as previously described [25], and used within the third passage. BJ5ta were cultured in DMEM (Euroclone)/Medium 199 (Gibco), supplemented with 10% FBS (ECS0183L, Euroclone), Stable Glutamine (Euroclone), 10 μg/mL hygromycin B (Invitrogen) and antibiotics (Euroclone). HMLE were cultured in DMEM/Ham’s F12 medium (Euroclone), supplemented with 0.05 mg/mL of Bovine Pituitary Extract (Gibco), Stable Glutamine (Euroclone), antibiotics (ECB3001D, Euroclone), 10 μg/mL Insulin (Sigma), 0.5 μg/mL Hydrocortisone (Sigma), and 10 ng/mL Epidermal Growth Factor (Cell Guidance System). MCF10A and MDA-MB231 were authenticated by STR genotyping and were tested for mycoplasma weekly. For all experiments, cells were cultured for less than a month after thawing.

*Growing cells as monolayers.* All cell models were plated at different concentrations: MCF10A: sparse = 2500 cells/cm^2^; subconfluent = 7000 cells/cm^2^; confluent = 60,000 cells/cm^2^; hyperconfluent = 90,000 cells/cm^2^. RWPE-1: sparse = 3000 cells/cm^2^; subconfluent = 21,000 cells/cm^2^; confluent = 59,000 cells/cm^2^; hyperconfluent = 100,000 cells/cm^2^. NIH-3T3: sparse = 1000 cells/cm^2^; confluent = 18,000 cells/cm^2^. BJ5ta: sparse = 1100 cells/cm^2^; confluent = 35,000 cells/cm^2^; HUVEC: sparse = 2000 cells/cm^2^; confluent = 47,000 cells/cm^2^. MDA-MB231: sparse = 5000 cells/cm^2^; subconfluent =16,000 cells/cm^2^; confluent = 70,000 cells/cm^2^. After 48 h, cells were photographed and processed for subsequent analysis. Images were captured using a Primovert KMAT microscope (Zeiss, Oberkochen, Germany).

*RNA interference.* Cells were cultured for 48 h, and then transfected with 40 nM siRNA oligonucleotides, using Lipofectamine^®^ RNAiMax (Invitrogen, Carlsbad, CA, USA) following the manufacturer’s instructions. siRNAs used are listed in Table 1.

For DAB2IP silencing we used a mixture of siDAB2IP A and siDAB2IP B, both robustly validated in our previous studies. After 6 h of transfection, the medium was changed. In all experiments, cells were silenced at least for 72 h, in order to obtain proper silencing of the target gene.

*Plasmids.* myc-hDAB2IP was amplified from pCMV-myc-hDAB2IP (kindly provided by Sidney Yu) and cloned in pLPC-puromycin resistant retroviral vector. pBABE puromycin-resistant empty vector, pBABE-hYAP-wt (puromycin resistant) and pBABE-hYAP-5SA (puromycin resistant) were kindly provided by Prof. Del Sal.

*Viral transduction.* For retroviruses production, Phoenix-GP packaging cells (CRL-3215, ATCC, Manassas, VA, USA) were transfected with the packaging plasmid and the plasmid construct of interest, using the standard calcium-phosphate method. After 8 h, the medium was changed and cells were incubated at 37 °C. After 48 h, the supernatants containing viral particles were filtered (0.45 μm filter) and supplemented with 10% FBS and polybrene (8 μg/mL). The culture medium of target cells growing at the low confluence (≈30–40%) was replaced by the appropriate viral supernatant and incubated at 37 °C for 24 h. Cells were selected with 0.5 μg/mL puromycin (Sigma) and kept under selection for the entire experiment. Monoclonal cell lines were obtained by limiting dilution and were selected based on the DAB2IP protein level detected by immunoblot and immunostaining.

*Cell detachment***.** To detach cells from the tissue culture plate, MCF10A cells were washed twice with PBS, then treated from 15 min to 1 h with 0.05% Trypsin-EDTA (15400054, Gibco), for 3 h with 10 mM EGTA (E4378, Sigma), or 3 h with 10 mM DTT (Sigma). After treatment, where applicable, cells were washed with PBS and a complete culture medium was added for the indicated time.

*Cytoskeleton drugs.* MCF10A cells were treated with 0.5 µM Latrunculin A (10010630, Cayman Chemical, Ann Arbor, MI, USA) for 4 h, with 10 µM Cytochalasin D (C2618, Sigma) for 4 h and with 50 µM Blebbistatin (B0560, Sigma) for 24 h. The ATP-competitive inhibitor of focal adhesion kinase (FAK) PF573228 (S2013, Selleck-chem, Houston, TX, USA), was used at 10 µM for 6 h. DMSO (Sigma) was used as a negative control.

### 2.2. Protein Analysis

Total cell extracts were prepared in RIPA buffer without SDS (150 mM NaCl, 50 mM Tris-HCl pH8, 1 mM EDTA, 1% NP-40, 0.5% Na-deoxycholate) supplemented with 1 mM PMSF, and 10 μg/mL CLAP, 5 mM NaF, and 1 mM Na_3_VO_4_. Protein concentration was determined with BioRad Protein Assay Reagent (500–0006, Bio-Rad, Hercules, CA, USA). Lysates were resolved by SDS/PAGE and transferred to nitrocellulose (Millipore, Burlington, MA, USA). Western blot analysis was performed according to standard procedures using the following primary antibodies: DAB2IP (A302-440A, Bethyl, Montgomery, TX, USA), E-cadherin (BD610182, BD Bioscience, Franklin Lakes, NJ, USA), FAK (sc-558, Santa Cruz, Dallas, TX, USA), phospho-FAK (8556s, Cell Signaling Technology, Danvers, MA, USA), YAP (sc-101199, Santa Cruz), phospho-YAP (4911s, Cell Signaling Technology), HSP90 (sc-13119, Santa Cruz), GAPDH (sc-32233, Santa Cruz), and c-myc (9E10, sc-40, Santa Cruz). HRP-conjugated anti-mouse (A90-516P) and anti-rabbit (A120-201P) IgG (Bethyl) were used as secondary antibodies. Bands were detected on autoradiographic film (GE Healthcare, Chicago, IL, USA) using Liteablot Extent chemiluminescent substrate (Euroclone) or Pierce™ ECL Western Blotting Substrate (Thermo Fisher Scientific, Waltham, MA, USA). Original data can be found at Supplementary File S1, S2 and Table S1.

### 2.3. RNA Extraction and RT-qPCR

Total RNA was extracted using TriFast II (Euroclone) following the manufacturer’s instructions. Purified RNA samples were quantified using an Eppendorf BioPhotmeter D30 and an Eppendorf μCuvette (Eppendorf, Hamburg, Germany). For RNA expression analysis, 0.5 μg of total RNA was reverse-transcribed using the iScript™ Advanced cDNA Synthesis Kit (BioRad, Hercules, CA, USA). A CFX Connect™ Real-Time PCR System (BioRad) was used, using Itaq UniversSYBR Green (BioRad), following the manufacturer’s instructions. Primers are listed in Table 2.

### 2.4. Immunofluorescence Microscopy

Cells were cultured for 48 h on coverslips previously incubated for 5 min with 1 μg/mL Poly-L-Lysine (Sigma). Then, cells were fixed for 20 min with 4% PFA (Sigma), and permeabilized with 0.1% Triton X- 100 (Sigma) for 10 min. After 30 min blocking with 3% BSA (Albumin fraction V, Sigma), cells were incubated with the following primary antibodies for 3 h at room temperature: DAB2IP (A302-440A, Bethyl), YAP/TAZ (sc-101199, Santa Cruz) and c-myc (sc-40, Santa Cruz). Primary antibodies were then recognized through 1 h incubation with anti-mouse or anti-rabbit Alexa Fluor^®^ 488/568 conjugated fluorescent secondary antibodies (A11004, A11001, A11011 and A11008, Invitrogen). Alternatively, a 1 h incubation step with Rhodamine phalloidin (R415, Invitrogen) was used to label actin filaments. Nuclei were labeled with Hoechst 33342 (Thermo Fisher Scientific), and then each coverslip was mounted with ProLong Gold antifade reagent (Invitrogen). Images were captured using a Leica DM4000B epifluorescence microscope at 40× magnification.

To determine YAP subcellular localization, images were analyzed by counting the percentage of cells with YAP into the nucleus, cytoplasm, or both cell compartments over total cell nuclei, in 20 random microscope fields.

The images for quantitation of cell density (nuclei/area) and nuclear areas were acquired using an inverted Nikon Ti microscope equipped with a motorized xyz table, perfect focus system and 20× objective; 4–10 images were randomly collected for each coverslip in order to avoid any user bias selection. Images were analyzed using the cell count and morphometric modules present in the NIS-Elements software (version 4.30, Nikon, Minato city, Tokyo, Japan). Nuclei were selected using a common threshold procedure for all images. As a morphological descriptor, the area was collected for each nucleus. Graphic presentation and statistics were performed in Prism 9. To analyze cytoskeleton disassembly after drug treatments, z-stack images were captured on a Zeiss lattice SIM Elyra7 structured illumination microscope, using a 63× oil objective, default lattice width for each laser line and 13 phases of illumination. Super-resolution reconstruction of the z-stacks was achieved using default parameters for the SIM module, before maximal intensity projection.

### 2.5. BrdU Incorporation Assay

Cells were plated on Poly-L-Lysine coated coverslips, and cultured for 48 h before being labeled by adding 20 μM BrdU (B5002 Sigma) for 2 h. After BrdU incorporation cells were fixed, permeabilized, and DNA was denatured with 50 mM NaOH. BrdU was detected by indirect immunofluorescence using a specific anti-Bromo-deoxyuridine monoclonal antibody (clone BU-1-Amersham). Images were captured using a Leica DM4000B epifluorescence microscope (Leica, Wetzlar, Germany) at 10× magnification. Proliferation was scored by counting BrdU-positive cells over total cell nuclei, in 20 random microscope fields.

### 2.6. AFM Force Spectroscopy

Force spectroscopy analysis of cells was carried out by using a Smena AFM (NT-MDT Co., Moscow, Russia) mounted on an inverted fluorescence microscope (Nikon Eclipse Ti-U). MCF10A cells were cultured on Poly-L-Lysine coated glass coverslips for 48 h, and then fixed for 20 min with 4% PFA (Sigma). To visualize nuclei, cells were stained with Hoechst 33342 solution (Thermo Fisher Scientific) for 10 min. Cells were analyzed with the AFM in contact mode in a liquid solution (PBS). For the measurement, a silicon spherical tip with a diameter of 20 µm (Tip: Nova Scan cantilever, k = 0.064 N/m) was used, in order to collect the global stiffness of each cell. A force (SetPoint) of <1 nN, a gain of 1 or 2, an indentation rate of 2 µm/s (low enough to avoid hydrodynamic effects) and an indentation of −500 nm (~10% of the total cell height) were used. For each sample, 30–60 cells were analyzed. Elastic modulus values (E), in kPa, were determined by fitting the obtained force-displacement curves with the Hertz model by using AtomicJ^®^ 2.3.1 software.

### 2.7. Luciferase Reporter Assay

Cells cultured for 48 h were transiently transfected with 250 ng/cm^2^ of pGL3-8xGTIIC-Lux reporter using Lipofectamine^®^ LTX (Invitrogen), following the manufacturer’s instructions. pGL3-8xGTIIC-Lux is a reporter with Luciferase (*P. pyralis*) under the control of a TEAD-responsive promoter (Dupont et al., 2011 [10]); 25 ng/cm^2^ of CMV-Renilla plasmid were transfected in order to normalize for transfection efficiency. After 24 h of transfection, cells were lysed in Passive Lysis Buffer (Promega, Madison, WI, USA); lysates were analyzed using Dual-luciferase Reporter Assay System Kit (Promega, E1910), on a TD-20/20 Luminometer (Turner Design, San Jose, CA, USA). Each sample was transfected in triplicate and each experiment was repeated three times independently.

### 2.8. Statistical Analysis

In all graphs, data are expressed as mean ± SD of at least three independent experiments, except when otherwise indicated. Differences were analyzed by Student’s *t*-test using Prism 9 (GraphPad). *p*-values < 0.05 were considered significant.

## 3. Results

### 3.1. DAB2IP Protein Levels Change with Cell Density in 2D Cultures

**Figure 1:**
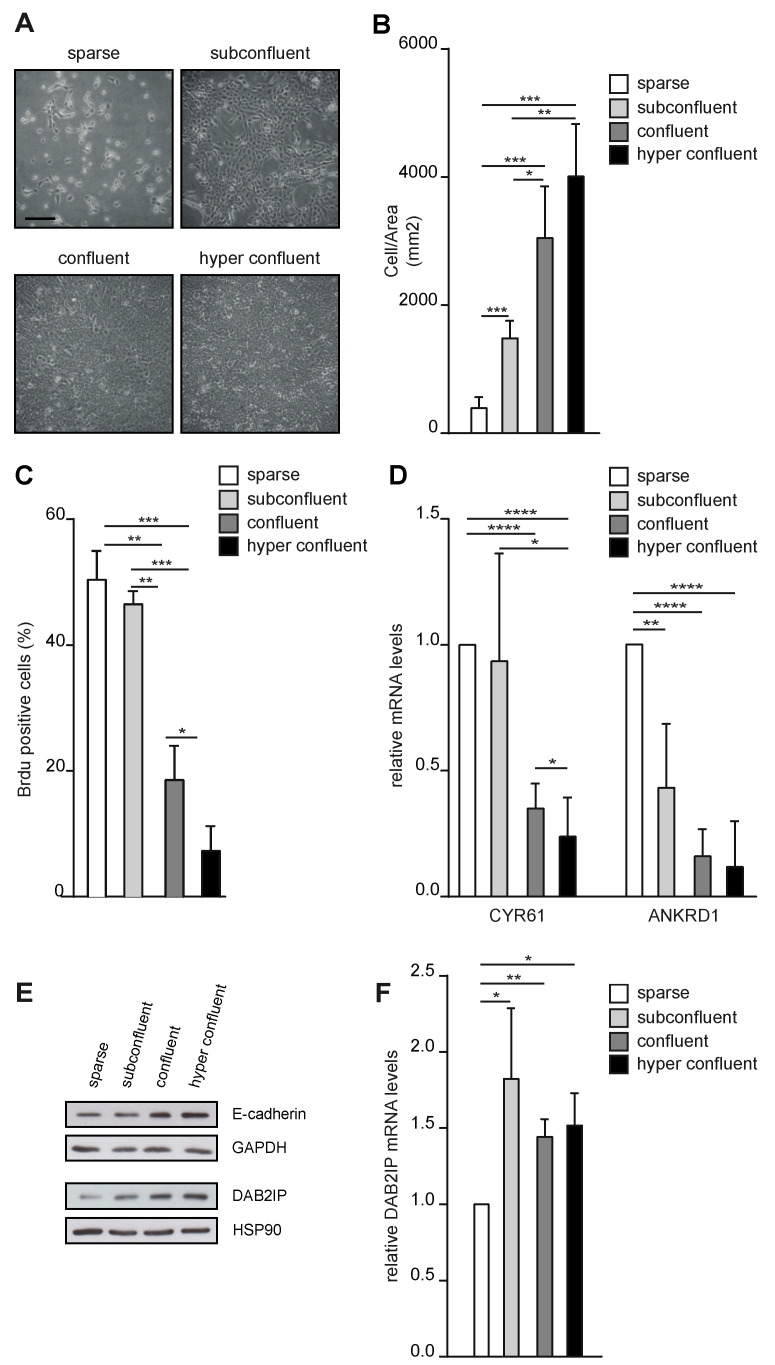
DAB2IP protein levels change with cell density in monolayer cultures. (**A**) Representative images of MCF10A cells seeded at different concentrations (sparse = 2500 cells/cm^2^; subconfluent = 7000 cells/cm^2^; confluent = 60,000 cells/cm^2^; hyperconfluent = 90,000 cells/cm^2^) and photographed after 48 h. Scale bar = 200 µm. (**B**–**F**) Increasing cell density correlates with reduced cell proliferation, reduced YAP/TAZ transcriptional activity, increased E-cadherin levels, and DAB2IP protein levels. MCF10A cells were cultured as described in (**A**) before subsequent analyses. (**B**) Cells were fixed, and nuclei were labeled with Hoechst and counted. Graph indicates the number of nuclei in a square area of 1 mm^2^ (mean ± SD; n = 4; * *p* <0.05, ** *p* < 0.01, *** *p* < 0.001). (**C**) Cells were labeled with BrdU for 2 h before fixation and immunostaining with an anti-BrdU antibody; nuclei were counterstained with Hoechst. Graphs summarize the percentage of BrdU positive nuclei (mean ± SD; n = 3; * *p* < 0.05, ** *p* < 0.01, *** *p* < 0.001). (**D**) Total RNA was extracted and expression of CYR61 and ANKRD1 was measured by RT-qPCR. Data were normalized on histone H3 (mean ± SD; n = 4; * *p* <0.05, ** *p* < 0.01, **** *p* < 0.0001). (**E**) Total cell lysate was analyzed by Western blot to detect E-cadherin and DAB2IP. GADPH and HSP90 were blotted as loading controls. (**F**) Expression of DAB2IP was measured by RT-qPCR as in D (mean ± SD; n = 3; * *p* < 0.05, ** *p* < 0.01).

To explore if DAB2IP levels are regulated by cell density, we seeded MCF10A mammary epithelial cells at different concentrations. We aimed to achieve the maximum density that would preserve a monolayer distribution—from now on confluency, or max 2D density (Figure 1A). Cell confluency was monitored qualitatively by checking the free space between cells and quantified by counting the number of nuclei per area (Figure 1B). We analyzed DNA synthesis by BrdU incorporation, confirming that confluency correlated with a significant reduction in cell proliferation (Figure 1C). Next, we assessed the activity of the co-transcriptional factors YAP and TAZ, which are sensitive to contact inhibition [26]. As expected, YAP/TAZ target genes CYR61 and ANKRD1 were downregulated at confluency (Figure 1D). Finally, the increase in cell density was paralleled by a gradual accumulation of E-cadherin (Figure 1E) [27,28].

Under these conditions, DAB2IP protein levels increased gradually from sparse to confluent cells (Figure 1E). This pattern of expression was confirmed in other cell lines, including endothelial cells and fibroblasts (Figure S1A–D), suggesting that regulation of DAB2IP by confluency is a general mechanism, shared by different cell types. To understand the basis of this regulation, we analyzed DAB2IP mRNA by RT-qPCR; we observed a moderate but significant downregulation in sparse cells, but no relevant differences between other culture conditions, thus, not mirroring variations in DAB2IP protein levels (Figure 1F). This suggests that low cell density could repress DAB2IP transcription, while different degrees of confluency could regulate DAB2IP at the post-transcriptional level.

### 3.2. DAB2IP Levels Are Reduced When Cells Are Detached from the Substrate or Lose Contact with Adjacent Cells

**Figure 2:**
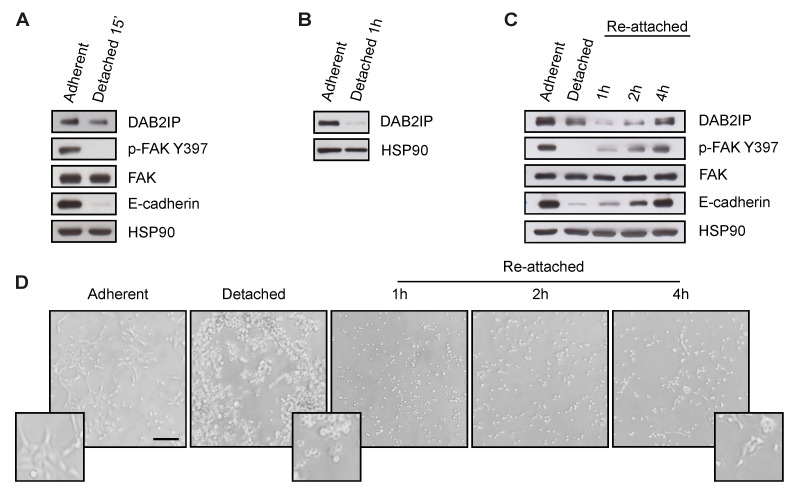
Cell adhesion to the substrate sustains DAB2IP protein levels. (**A**) Cell detachment decreases DAB2IP protein levels. MCF10A cells seeded on plastic (7000 cells/cm^2^) were cultured for 48 h before being detached by Trypsin/EDTA treatment. After 15 min, adherent and detached cells were collected and lysed. DAB2IP, p-FAK Y397, total FAK and E-cadherin were detected by immunoblotting, with HSP90 as loading control. (**B**) Lack of attachment decreases DAB2IP protein levels. Cells were seeded at high density (60,000 cells/cm^2^) and cultured for 48 h until confluence, before being detached by Trypsin/EDTA treatment. Detached cells were kept in suspension for 1 h, then adherent and detached cells were collected and lysed. DAB2IP was detected by immunoblotting, with HSP90 as a loading control. (**C**,**D**) Cell attachment increases DAB2IP protein levels. MCF10A cells were cultured and detached by Trypsin/EDTA, as in (**A**). After 15 min, cells were seeded in Petri dishes and kept in culture for the indicated times. (**C**) Adherent, detached and re-attached cells were collected and lysed. DAB2IP, p-FAK Y397, total FAK and E-cadherin were detected by immunoblotting, with HSP90 as loading control. (**D**) Representative pictures of adherent, detached and re-attached cells photographed before lysis (scale bar = 200 µm).

We asked whether DAB2IP levels would decrease when cell interactions are disrupted. We first detached subconfluent MCF10A cells from the culture dish by Trypsin/EDTA treatment. We observed a significant reduction of DAB2IP protein, along with the expected reduction of E-cadherin levels and FAK activity (Figure 2A). DAB2IP downregulation was even more striking when the experiment was done with confluent MCF10A detached and kept in suspension for 1 h (Figure 2B). When detached cells were re-plated on plastic, there was a progressive recovery of DAB2IP protein, as well as E-cadherin levels and FAK activity (Figure 2C,D), suggesting that cell interactions and/or mechanical activation support DAB2IP expression.

**Figure 3:**
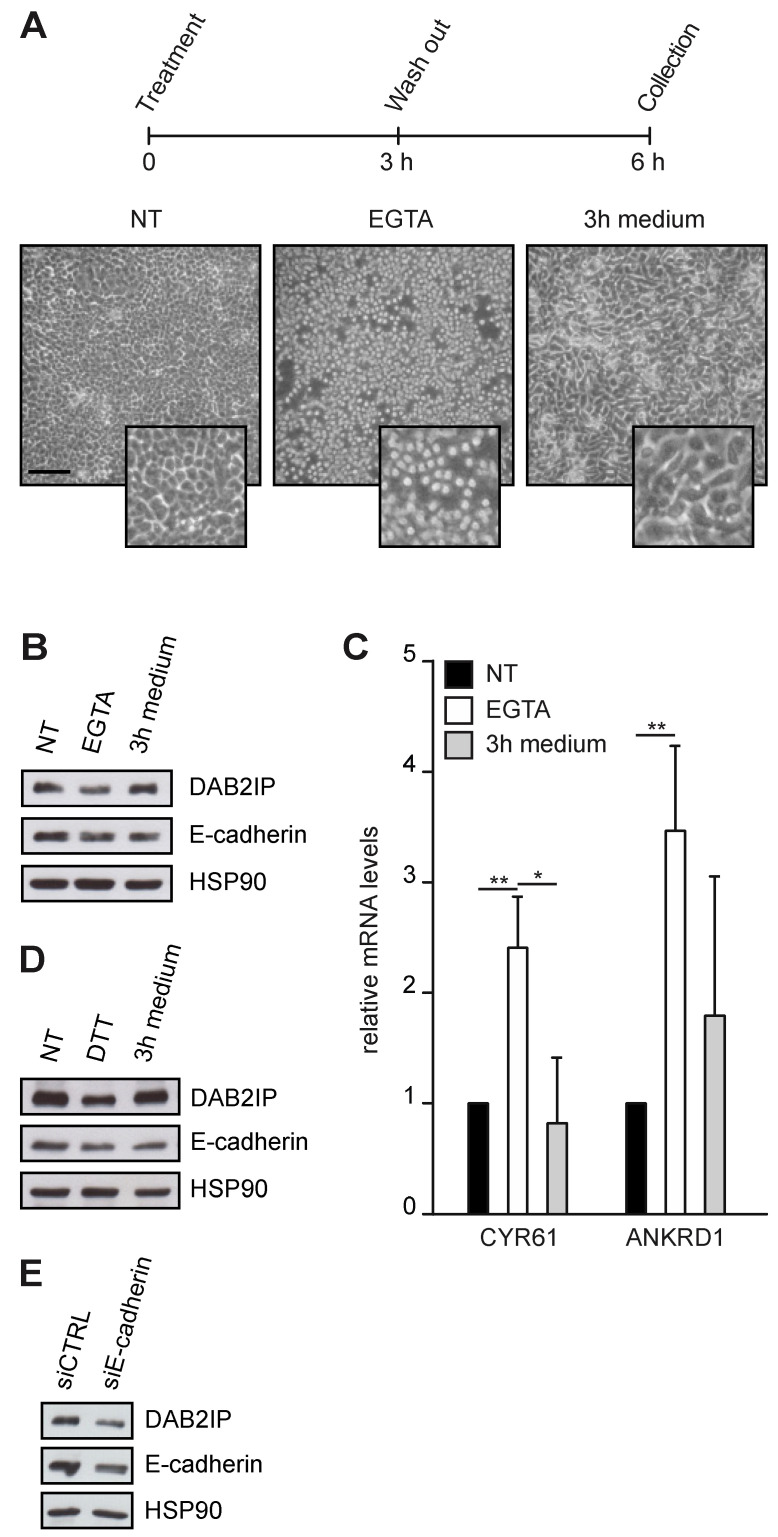
Cell–cell interactions sustain DAB2IP protein levels. (**A**–**C**) DAB2IP protein levels and YAP/TAZ activity are reversibly affected by EGTA treatment. MCF10A cells were seeded at high density (60,000 cells/cm^2^) and cultured for 48 h until confluence. Cells were treated with 10 mM EGTA for 3 h, then fresh medium was added for an additional 3 h. (**A**) Schematic protocol and representative pictures of cells at the indicated time points (scale bar = 200 µm). (**B**) DAB2IP and E-cadherin levels were detected by immunoblotting, with HSP90 as a loading control. (**C**) Expression of CYR61 and ANKRD1 was measured by RT-qPCR. Data are normalized on histone H3 (mean ± SD; n = 3; * *p* < 0.05, ** *p* < 0.01). (**D**) DAB2IP protein levels are reversibly affected by DTT treatment. MCF10A cells were cultured and treated exactly as in A, using 10 mM DTT instead of EGTA. DAB2IP and E-cadherin levels were detected by immunoblotting, with HSP90 as a loading control. (**E**) E-cadherin depletion reduces DAB2IP protein in confluent cells. MCF10A were transfected with the indicated siRNAs, then seeded (60,000 cells/cm^2^) and cultured for an additional 48 h to confluent condition. DAB2IP and E-cadherin levels were detected by immunoblotting, with HSP90 as a loading control.

We next treated confluent MCF10A with EGTA, a chelator of extracellular Ca^2+^ that breaks the homophilic binding between cadherins. Cells remained attached to the substrate, but dissociated from adjacent cells, adopting a round shape. When EGTA was removed and replaced with a Ca^2+^-containing medium, cells returned to a normal shape with the re-assembly of cell junctions (Figure 3A). Using this protocol, we found that disruption of cell–cell junctions reduced DAB2IP protein levels, which were restored once junctions were re-established (Figure 3B). Transcription of YAP/TAZ target genes CYR61 and ANKRD1 was induced by EGTA treatment and repressed after cell junctions reassembly—confirming that YAP/TAZ are inhibited by cell–cell contact (Figure 3C). Our data cannot exclude that EGTA also affects DAB2IP levels by alteration of some Ca^2+^-dependent pathways [29]; for this reason, we repeated the experiment using DTT, which prevents cadherin interactions by reducing disulfide bridges [30]. Like EGTA, DTT also caused a decrease in DAB2IP protein levels, which were rescued after drug washout (Figure 3D).

Since both treatments do not significantly interfere with focal adhesions, these results imply that cell–cell contacts have a substantial role in DAB2IP regulation. To test this concept, we evaluated the impact of E-cadherin knockdown; in line with previous experiments, transfection of a siRNA against E-cadherin in confluent MCF10A cells decreased DAB2IP protein levels (Figure 3E). E-cadherin depletion reduced DAB2IP levels also in subconfluent cells (Figure S2A), increasing transcription of YAP/TAZ targets CYR61 and ANKRD1 (Figure S2B). We noticed that depletion of E-cadherin, as well as EGTA treatment, caused a modest reduction of DAB2IP mRNA (Figure S2C,D), suggesting that cell–cell junctions may directly or indirectly affect its transcription or RNA stability.

### 3.3. Cytoskeletal Tension Sustains DAB2IP Levels in Low-Density Conditions but Is Not Required in Confluent Cells

**Figure 4:**
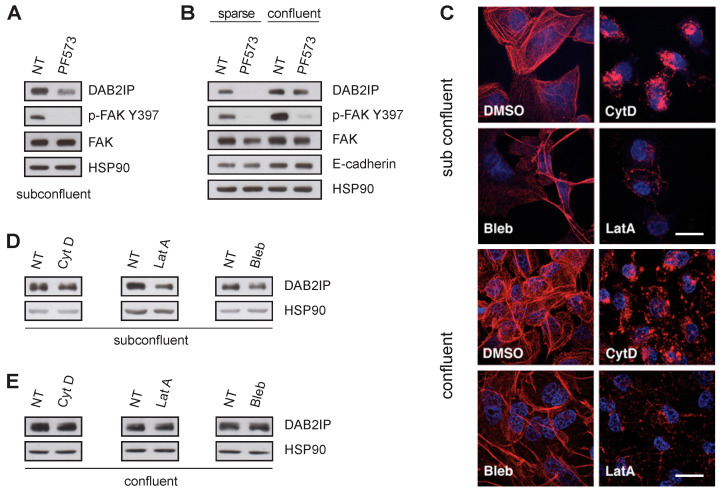
Mechanical tension of the actin cytoskeleton is not necessary to sustain DAB2IP protein levels in confluent cells. (**A**,**B**) FAK inhibition reduces DAB2IP protein levels in subconfluent cells, but not in confluent monolayers. MCF10A were seeded and cultured for 48 h to a subconfluent, sparse or confluent condition, as in Figure 1, and then treated with 10 µM PF573228 (PF573) for 6 h before lysis. DAB2IP, p-FAK Y397, total FAK and E-cadherin were detected by immunoblotting, with HSP90 as loading control. (**C**–**E**) Disruption of actin cytoskeleton reduces DAB2IP protein levels in subconfluent cells, but not in confluent monolayers. MCF10A were seeded and cultured to subconfluent or confluent conditions, and then were exposed to Cytochalasin D (Cyt D, 10 µM for 4 h), Latrunculin A (LatA, 0.5 µM for 4 h), or Blebbistatin (Bleb, 50 µM for 24 h); then, cells were processed for immunofluorescence or Western blot. (**C**) Representative images of treated and control cells. F-actin was visualized with rhodamine phalloidin (red). Nuclei were labeled with Hoechst (blue). Scale bar = 20 µm. (**D**,**E**) DAB2IP was analyzed by immunoblotting after cells were treated with the indicated drugs in subconfluent conditions (**D**) or as a confluent monolayer (**E**). HSP90 was blotted as a loading control.

Cell junctions mechanically associate the cytoskeleton with the surrounding environment, controlling specific biochemical and transcriptional responses. We, therefore, evaluated the role of the actin cytoskeleton in the observed DAB2IP regulation. We first treated MCF10A cells with the FAK inhibitor PF573228 (PF573) which decreases focal adhesion turnover and destabilizes actin filaments, lowering the tensional state of the cytoskeleton [31]. Notably, treatment with PF573 reduced DAB2IP in subconfluent and sparse cells, but not in cells at high density (Figure 4A,B), suggesting that FAK activity is important to sustain DAB2IP levels in low density, but is dispensable in confluent cells.

Next, we treated MCF10A with cytoskeleton inhibitors Latrunculin A, Cytochalasin D and Blebbistatin (see methods for details); drug effects on microfilaments were monitored by phalloidin staining (Figure 4C). In subconfluent cells, DAB2IP protein decreased after treatment, indicating a dependency on cytoskeletal organization and tension (Figure 4D). However, in high-density conditions, these drugs had non-significant effects on DAB2IP levels (Figure S3A and Figure 4E), suggesting that other inputs must be active in confluent cells. Notably, depletion of E-cadherin caused DAB2IP downregulation regardless of microfilaments’ integrity (Figure S3B,C), confirming that DAB2IP expression in confluent cells is mostly dependent on cell–cell junctions.

Importantly, similar results were obtained in the triple-negative breast cancer cell line MDA-MB-231, where disruption of the actin cytoskeleton reduced DAB2IP in low density, but had negligible effects in high-density conditions (Figure S3D,E).

Together, these data suggest that DAB2IP levels can be regulated by cytoskeletal tension (developed at cell–ECM and/or cell–cell junctions), but in confluent cells, DAB2IP is controlled by other signals, likely dependent on cell–cell interactions but unrelated to FAK activity or actomyosin fiber contraction.

### 3.4. DAB2IP Depletion Alters the Morphology of Epithelial Cells Grown at Confluency

**Figure 5:**
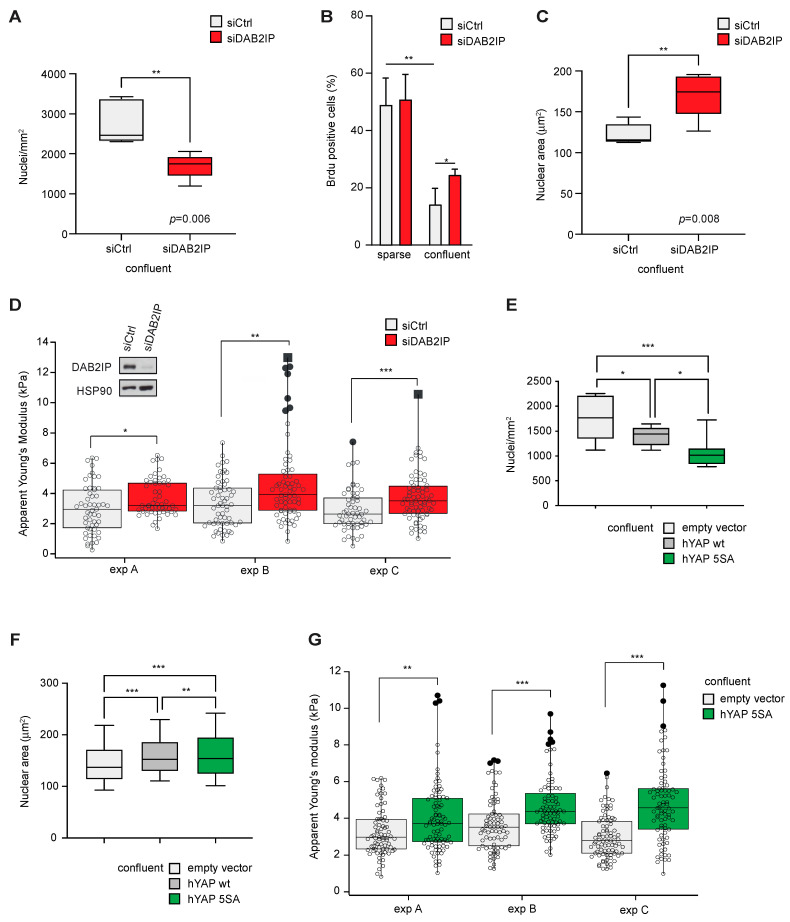
DAB2IP depletion affects the morphology, proliferation and stiffness of confluent cells. (**A**) DAB2IP depletion reduces maximal cell density in a confluent monolayer. MCF10A cells were transfected with the indicated siRNAs (40 nM) for 24 h, then seeded at high density (60,000 cells/cm^2^) and cultured for an additional 48 h to full confluency. Cells were fixed and nuclei were labeled with Hoechst. Graph summarizes the average number of nuclei per area in at least 4 microscope fields (mean ± SD; n = 5; ** *p* < 0.01). (**B**) DAB2IP depletion increases cell proliferation in high-density cultures. MCF10A cells were silenced as in (**A**), and seeded at low or high density. After 48 h, cells were labeled with BrdU for 2 h before fixation and immunostaining with an anti-BrdU antibody; nuclei were counterstained with Hoechst. Graphs summarize the percentage of BrdU positive nuclei (mean ± SD; n = 3; * *p* < 0.05, ** *p* < 0.01). (**C**) DAB2IP depletion increases the nuclear size of confluent cells. MCF10A cells were cultured and labeled exactly as in (**A**). Graph indicates the average nuclear area of at least 1100 cells in 4 microscope fields (mean ± SD; n = 5 ** *p* < 0.01). (**D**) DAB2IP depletion increases the stiffness of confluent cells. MCF10A cells were cultured and labeled exactly as in A. Cell rigidity was measured using atomic force microscopy (AFM) as detailed in the methods. At least 50 cells were measured per sample; black dots indicate outliers, and black squares are far outliers. Data refer to three independent experiments (* *p* < 0.05, ** *p* < 0.01, *** *p* < 0.001). (**E**–**G**) Constitutive YAP overexpression in MCF10A recapitulates the phenotypes observed after DAB2IP knockdown. MCF10A stably overexpressing hYAP wt, hYAP 5SA, or an empty vector were cultured to maximal confluency and labeled as in (**A**). (**E**) Graph indicates the number of nuclei per area in at least 4 microscope fields (mean ± SD; n = 3; * *p* < 0.05, *** *p* < 0.001). (**F**) Graph indicates the average nuclear area of at least 800 cells in 4 microscope fields (mean ± SD; n = 3; ** *p* < 0.01, *** *p* < 0.001). (**G**) Cell rigidity was measured using atomic force microscopy (AFM) as in (**D**). At least 50 cells were measured per sample; black dots indicate outliers. Data refer to three independent experiments (** *p* < 0.01, *** *p* < 0.001).

Given its upregulation with cell density, we analyzed the impact of DAB2IP depletion in different confluence conditions. Subconfluent MCF10A cells display luminal ductal features [32]; upon DAB2IP knockdown, they change their morphology, tend to be more spread on the culturing surface and acquire a spindle-like shape (Figure S4A—left panels). This phenotype is in line with features of EMT and invasive behavior observed by others in DAB2IP-depleted MCF10A [15]. In confluent monolayers, DAB2IP-depleted MCF10A cells are morphologically similar to controls but less tightly packed (Figure S4A, right panels). Specifically, DAB2IP knockdown cells reproducibly displayed a lower number of nuclei per area (Figure 5A and Figure S4B), and an increased proliferation index as measured by BrdU incorporation (Figure 5B). Notably, confluent DAB2IP knockdown cells also showed increased nuclear area (Figure 5C and Figure S4B). Such features may suggest a change in the mechanical properties of confluent cells; analysis based on Atomic Force Microscopy (AFM) confirmed that DAB2IP-depleted cells have a higher elastic module than controls, indicative of greater cell stiffness (Figure 5D).

Interestingly, similar effects have been described in epithelial cells after YAP hyperactivation [33]. We, therefore, analyzed MCF10A cells stably overexpressing YAP, or its constitutively active mutant YAP(5SA) [34]; these cells also display YAP/TAZ activity when grown to confluence (Figure S4C). Similar to DAB2IP knockdown cells, when grown at max. 2D density, YAP(5SA) overexpressing cells displayed reduced cell packing, larger nuclear area and greater stiffness (Figure 5E–G and Figure S4D). We conclude that in confluent cells the phenotype of DAB2IP knockdown is partially similar to that of YAP hyperactivation.

### 3.5. DAB2IP Contributes to YAP/TAZ Inhibition in Confluent Cells

**Figure 6:**
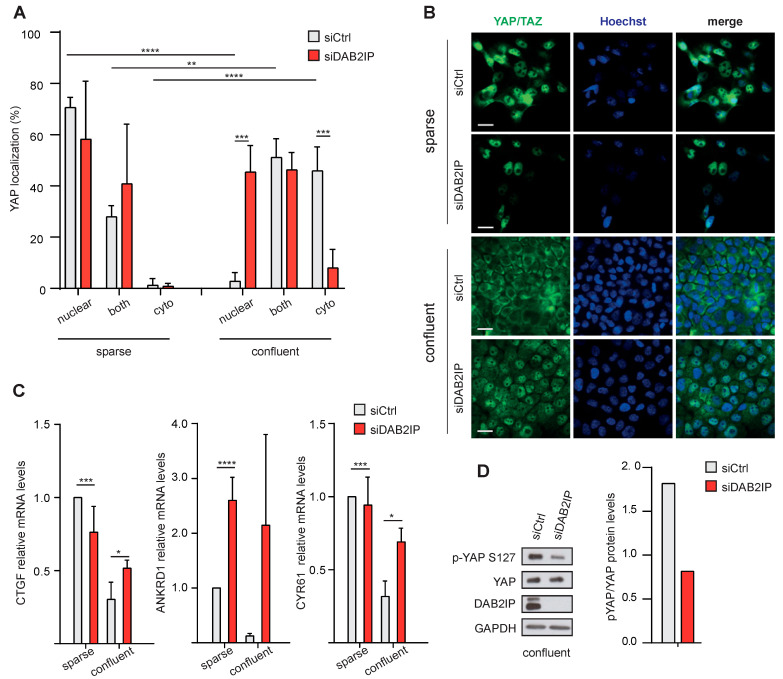
DAB2IP depletion allows YAP/TAZ activation in confluent cells. (**A**,**B**) DAB2IP depletion increases YAP/TAZ nuclear localization in confluent cells. MCF10A cells were transfected and seeded as described in Figure 5. The cellular distribution of YAP was analyzed by immunofluorescence. (**A**) Graph summarizes the percentage of cells with YAP staining in the nucleus, cytoplasm, or both compartments (mean ± SD; n = 4; ** *p* < 0.01, *** *p* < 0.001, **** *p* < 0.0001). (**B**) Representative images of YAP/TAZ (green) localization by immunofluorescence. Scale bar = 20 µm. (**C**) DAB2IP depletion increases the expression of YAP/TAZ target genes in confluent cells. MCF10A cells were treated as in A. Expression of CTGF, ANKRD1 and CYR61 was measured by RT-qPCR. Data were normalized on histone H3 (mean ± SD; n = 3; * *p* < 0.05, *** *p* < 0.001, **** *p* < 0.0001). (**D**) DAB2IP depletion reduces YAP Ser127 phosphorylation in confluent cells. MCF10A cells were cultured as in A. DAB2IP, phosphorylated (p-S127) and total YAP were detected by immunoblotting, with GAPDH as loading control. Protein bands were quantified and normalized to GAPDH by densitometry of autoradiography film (histogram on the right).

We next asked if DAB2IP could be involved in YAP/TAZ inhibition in MCF10A cells. As expected, in confluent control cells YAP was mainly cytoplasmic; under these conditions, DAB2IP knockdown induced YAP nuclear localization and transcription of YAP/TAZ target genes (Figure 6A–C). A similar effect on YAP/TAZ target genes was observed after DAB2IP depletion in confluent MDA-MB-231 cells (Figure S5A). Interestingly, in cells cultured at low density, DAB2IP knockdown did not affect YAP localization, or expression of YAP/TAZ target genes—with the exception of ANKRD1, which was upregulated also in sparse cells (Figure 6A–C).

Together, these experiments suggest that DAB2IP may modulate YAP/TAZ activity specifically in confluent cells.

Regarding mechanism, DAB2IP depletion reduced the inhibitory Ser127 phosphorylation of YAP in confluent MCF10A and MDA-MB-231 cells (Figure 6D and Figure S5B), suggesting that DAB2IP may directly or indirectly stimulate YAP kinases, or counteract YAP phosphatases.

**Figure 7:**
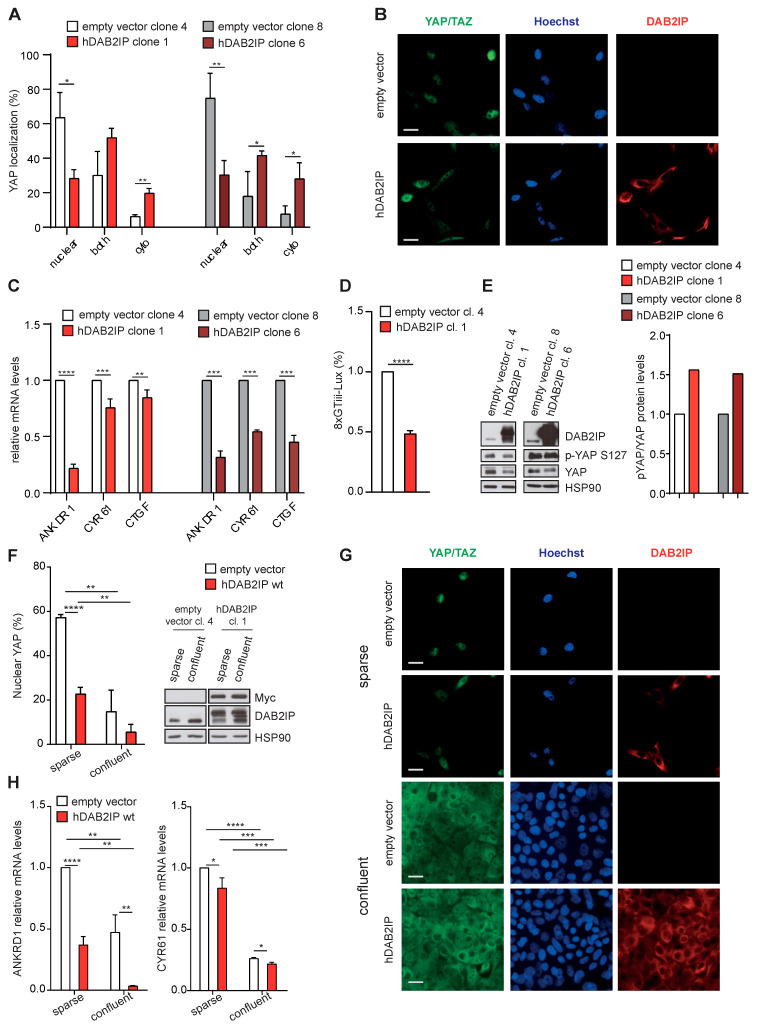
DAB2IP overexpression inhibits YAP/TAZ activity. (**A**,**B**) Expression of hDAB2IP induces YAP cytoplasmic retention. MCF10A cells stably expressing Myc-tagged hDAB2IP or a control empty vector (two different clones each), were seeded (7000 cells/cm^2^) and cultured for 48 h until subconfluent. YAP/TAZ localization was detected by immunofluorescence. (**A**) Graph summarizes the percentage of cells with YAP staining in the nucleus, cytoplasm, or both compartments (mean ± SD; n = 3; * *p* <0.05, ** *p* < 0.01). (**B**) Representative images of cells stained with antibodies against DAB2IP (red) and YAP/TAZ (green). Nuclei were stained with Hoechst (blue). Scale bar = 20 µm. (**C**,**D**) Stable expression of hDAB2IP inhibits YAP/TAZ transcriptional activity. MCF10A clones were cultured as in (**A**). (**C**) Expression of endogenous ANKDR1, CYR61 and CTGF was measured by RT-qPCR. Data were normalized on histone H3 (mean ± SD; n = 3; ** *p* < 0.01, *** *p* < 0.001, **** *p* < 0.0001). (**D**) The indicated cells were transfected with the 8xGTIIC-Lux reporter for 24 h, and YAP/TAZ activity was measured by Dual-luciferase assay (mean ± SD; n = 3; **** *p* < 0.0001). (**E**) Stable expression of hDAB2IP increases YAP Ser127 phosphorylation. MCF10A clones were cultured as in A. DAB2IP, phosphorylated (p-S127) and total YAP were detected by immunoblotting, with HSP90 as loading control. Protein bands were quantified and normalized to HSP90 by densitometry of autoradiography film (histogram on the right). (**F**–**H**) Stable expression of hDAB2IP inhibits YAP/TAZ nuclear translocation and transcriptional activity both in sparse and confluent cells. MCF10A clones were cultured for 48 h after seeding at low or high density (2500 cells/cm^2^ and 60,000 cells/cm^2^, respectively). (**F**,**G**) YAP/TAZ localization was analyzed by immunofluorescence as in (**A**). (**F**) Graph on the left indicates the percentage of cells with YAP/TAZ staining in the nucleus (mean ± SD; n = 3; * *p* < 0.05, ** *p* < 0.01, **** *p* < 0.0001). Expression of Myc-hDAB2IP and endogenous DAB2IP was checked by Western blot, with HSP90 as a loading control (right panel). (**G**) Representative images of cells stained with antibodies against DAB2IP (red) and YAP/TAZ (green). Nuclei were stained with Hoechst (blue). Scale bar = 20 µm. (**H**) Expression of ANKDR1 and CYR61 was measured by RT-qPCR. Data were normalized on histone H3 (mean ± SD; n = 3; * *p* < 0.05, ** *p* < 0.01, *** *p* < 0.001, **** *p* < 0.0001).

Since DAB2IP depletion appears to activate YAP/TAZ in confluent cells, a condition in which they are normally inhibited, we asked if DAB2IP overexpression would inhibit YAP/TAZ in culture conditions in which they are normally active. To this aim, we generated cell lines stably expressing hDAB2IP. Under standard culture conditions (i.e., subconfluent), hDAB2IP overexpression increased YAP cytoplasmic retention in two independent clonal cell lines (Figure 7A,B). Accordingly, YAP/TAZ target genes were also less expressed (Figure 7C).

Using a well-established YAP/TAZ responsive luciferase reporter [10], we confirmed that hDAB2IP overexpression reduced YAP/TAZ transcriptional activity in both stably and transiently transfected cells (Figure 7D and Figure S6). By immunoblotting, we detected increased YAP Ser127 phosphorylation in hDAB2IP overexpressing cells (Figure 7E). Together, these observations support the notion that DAB2IP contributes to YAP inhibition in confluent cells.

Finally, we analyzed the impact of hDAB2IP overexpression in cells cultured at very low or high density. As in subconfluent cultures, in sparse cells, hDAB2IP overexpression reduced YAP nuclear localization and transcription of YAP/TAZ target genes (Figure 7F–H). Less predictably, hDAB2IP also reduced YAP nuclear localization and expression of target genes in confluent monolayers, a condition in which YAP/TAZ activity is presumed to be minimal or null. This last observation implies that even in confluent cells there are biochemical inputs that drive YAP/TAZ activity, and DAB2IP may contribute to their physiological inhibition.

## 4. Discussion

The RasGAP and adaptor protein DAB2IP function as a negative modulator of several important signaling cascades [12,13]. By interacting with cytoplasmic transduction components, the stoichiometry of available DAB2IP protein in a cell can limit specific signaling events, thus affecting the corresponding biological response. Accordingly, DAB2IP downregulation or loss-of-function is predicted to amplify cellular responses to inputs with potentially oncogenic outcomes. Despite substantial knowledge about its pathological downregulation in cancer, very little is currently known about the regulation of DAB2IP in physiological conditions. Here, we analyzed non-transformed mammary epithelial cells and found that DAB2IP protein levels are modulated by cell density, being maximal in confluent monolayers. Loosening cell–cell or cell–ECM interactions reduced DAB2IP levels in tissue culture, suggesting that DAB2IP may be downregulated when epithelial integrity is perturbed in living tissues.

Cell density and substrate attachment are mechanical inputs sensed by cells via organization and tension of the actin cytoskeleton; intriguingly, DAB2IP levels are dependent on microfilaments in low density, but not in confluent cells (Figure 4). We hypothesize that two pathways can control DAB2IP levels: one linked to actomyosin tension, the other specifically reliant on cell–cell junctions’ integrity. Future studies will be aimed at testing this hypothesis.

When MCF10A lacking DAB2IP were grown to confluence, they displayed a reduced capacity to pack and squeeze (n. of cells/area) and showed larger nuclei and greater stiffness than control cells (Figure 5). The molecular bases of this phenotype remain to be clarified. Interestingly, we found that loss of DAB2IP has an effect on YAP (and perhaps TAZ). In fact, DAB2IP knockdown reduced YAP inhibitory phosphorylation in confluent cells, promoting its nuclear localization and transactivation of YAP/TAZ target genes. Conversely, DAB2IP overexpression increased YAP cytoplasmic retention in subconfluent cells, inhibiting YAP/TAZ-dependent transcription.

The morphological phenotype of DAB2IP knockdown could be the cause of increased YAP/TAZ activity. In fact, if DAB2IP depletion per se makes confluent cells larger and stiffer, this could activate YAP/TAZ by mechanosignaling. Alternatively, if DAB2IP depletion causes ectopic YAP/TAZ activation, this may be sufficient to induce the observed changes in cell stiffness and nuclear size, as we noted in YAP(5SA)-expressing MCF10A (Figure 5). Clearly, more detailed studies are required to uncover the cellular changes behind the morphological phenotype.

The mechanism by which DAB2IP negatively controls YAP remains to be defined; we deem unlikely a direct interaction, because we could not co-immunoprecipitate YAP and DAB2IP in our cell lines, and the two proteins were never co-purified in published protein–protein interaction screens (BioGrid version 4.4.220, https://thebiogrid.org/; IntAct release 243, https://www.ebi.ac.uk/intact/; accessed on 4 April 2023). It is possible that DAB2IP controls YAP by modulating one or more of its upstream regulators, either directly or indirectly. For instance, DAB2IP is a Ras-GAP and RTK-Ras signaling can activate YAP/TAZ by various mechanisms, including inhibition of Hippo pathway effectors [35], altered YAP turnover [36] and Rac1-dependent mechanosignaling [37,38]. DAB2IP also binds and inhibits PI3K [39], and a PI3K-PDK1 axis was shown to mediate YAP nuclear translocation in contact-inhibited serum-starved MCF10A cells treated with EGF [40]. Therefore, DAB2IP loss-of-function could facilitate YAP/TAZ activation by RTK-Ras or RTK-PI3K signaling.

DAB2IP is also a negative regulator of the canonical Wnt pathway: by recruiting PP2A phosphatase, it promotes GSK3b activation and β-catenin degradation [41]; by directly binding Rac1, it prevents β-catenin nuclear transport [42]. Notably, YAP and TAZ can bind to components of the β-catenin destruction complex and, at least under some circumstances, WNT signaling can lead to YAP/TAZ activation [6,34,43,44]. Therefore, DAB2IP loss-of-function could facilitate YAP/TAZ activation via the deregulation of the canonical WNT pathway.

Future work will be aimed at clarifying the mechanism by which DAB2IP contributes to YAP/TAZ inhibition in confluent cells.

## 5. Conclusions

Despite several unanswered questions regarding the mechanism, our experiments reveal two previously unreported features of DAB2IP biology that may have important implications for cancer. The first is that DAB2IP is downregulated when cells are detached or grown at low density; this suggests that DAB2IP levels would drop in pre-metastatic cells that are pushed out of an epithelium during tumor growth, and this would facilitate the aberrant signaling of oncogenic pathways such as RAS and NF-kB. The second is that DAB2IP contributes to YAP inhibition in confluent cells; this implies that transcriptional or post-transcriptional downregulation of DAB2IP would facilitate or enhance YAP/TAZ activation by mechanical inputs in precancerous epithelial cells, thus accelerating their evolution towards a tumorigenic fate. Collectively, our findings confirm that DAB2IP plays an important role in dampening oncogenic pathways in epithelial cells, including a previously unreported effect on YAP/TAZ, and suggest that DAB2IP may have a tumor-suppressive role in the early steps of metastasis, when alterations in tissue integrity and loosened cell–cell interactions can potentially reprogram cancer cells towards a fully invasive phenotype.

## Figures and Tables

**Table 1:** Sequence of siRNAs.

siRNA	Sequence	Purchased from
Control siRNA	Unknown	AllStars negative control (1027281, Qiagen, Venlo, The Netherlands)
siDAB2IP A	GGAGCGCAACAGUUACCUG	Eurofins (Luxembourg City, Luxembourg)
siDAB2IP B	GGUGAAGGACUUCCUGACA	Eurofins
siE-cadherin	GCAGAAAUUAUUGGGCUCUUU	Eurofins

**Table 2:** Sequence of PCR primers.

Target		Sequence
ANKRD1	Forward	5′-CACTTCTAGCCCACCCTGTGA-3′
	Reverse	5′-CCACAGGTTCCGTAATGATTT-3′
CTGF	Forward	5′-AGGAGTGGGTGTGTGACGA-3′
	Reverse	5′-CCAGGCAGTTGGCTCTAATC-3′
CYR61	Forward	5′-AGCCTCGCATCCTATACAACC-3′
	Reverse	5′-TTCTTTCACAAGGCGGCACTC-3′
DAB2IP	Forward	5′-CACATCACCAACCACTAC-3′
	Reverse	5′-TCCACCTCTGACATCATC-3′
YAP	Forward	5′-GCCGGAGCCCAAATCC-3′
	Reverse	5′-GCAGAGAAGCTGGAGAGGAATG-3′
H3	Forward	5′-GAAGAAACCTCATCG TTACAGGCCTGGT-3′
	Reverse	5′-CTG CAAAGCACCAATAGCTGCACTCTGGAA-3′

## Data Availability

No new datasets were created or analyzed in this study.

## Supplementary Materials

The following supporting information can be downloaded at: https://www.mdpi.com/article/10.3390/cancers15133379/s1, Figure S1: DAB2IP protein levels change with cell density in monolayer cultures, Figure S2: Cell-cell interactions affect DAB2IP expression, Figure S3: Cytoskeletal tension is not necessary to sustain DAB2IP protein levels in confluent cells, Figure S4: DAB2IP depletion or YAP overexpression modify the morphology of confluent cells, Figure S5: DAB2IP depletion allows YAP/TAZ activation in breast cancer MDA-MB-231 cells, Figure S6: DAB2IP overexpression inhibits YAP/TAZ activity. File S1: Original blots of Figure 1, Figure 2, Figure 3, Figure 4, Figure 6 and Figure 7. File S2: Original blots of Supplementary Figures S1–S6. Table S1: Quantification of all blots.
