# Supplementary material for: The Tumor Suppressor DAB2IP Is Regulated by Cell Contact and Contributes to YAP/TAZ Inhibition in Confluent Cells
Source: Cancers (Basel). 2023 Jun 27;15(13):3379. doi: 10.3390/cancers15133379 (PMC10340159; doi:10.3390/cancers15133379)
Supplement: Supplementary file 1 [file cancers-15-03379-s001.zip › File S1. Original blots Figures Rev.pdf]

Figure 1E: E-cadherin and GAPDH

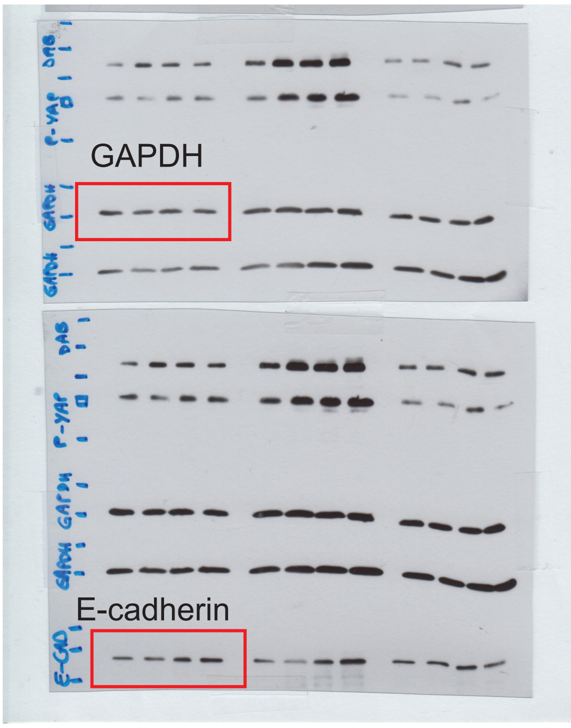

Figure 1E: DAB2IP and HSP90

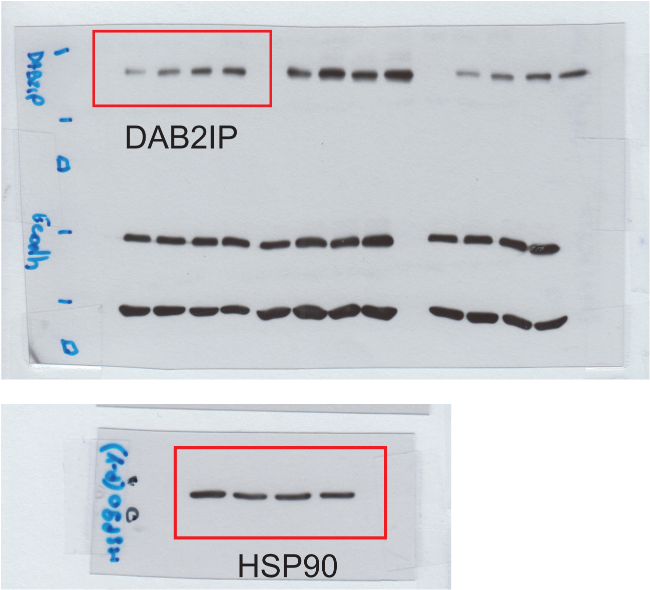

Figure 2A

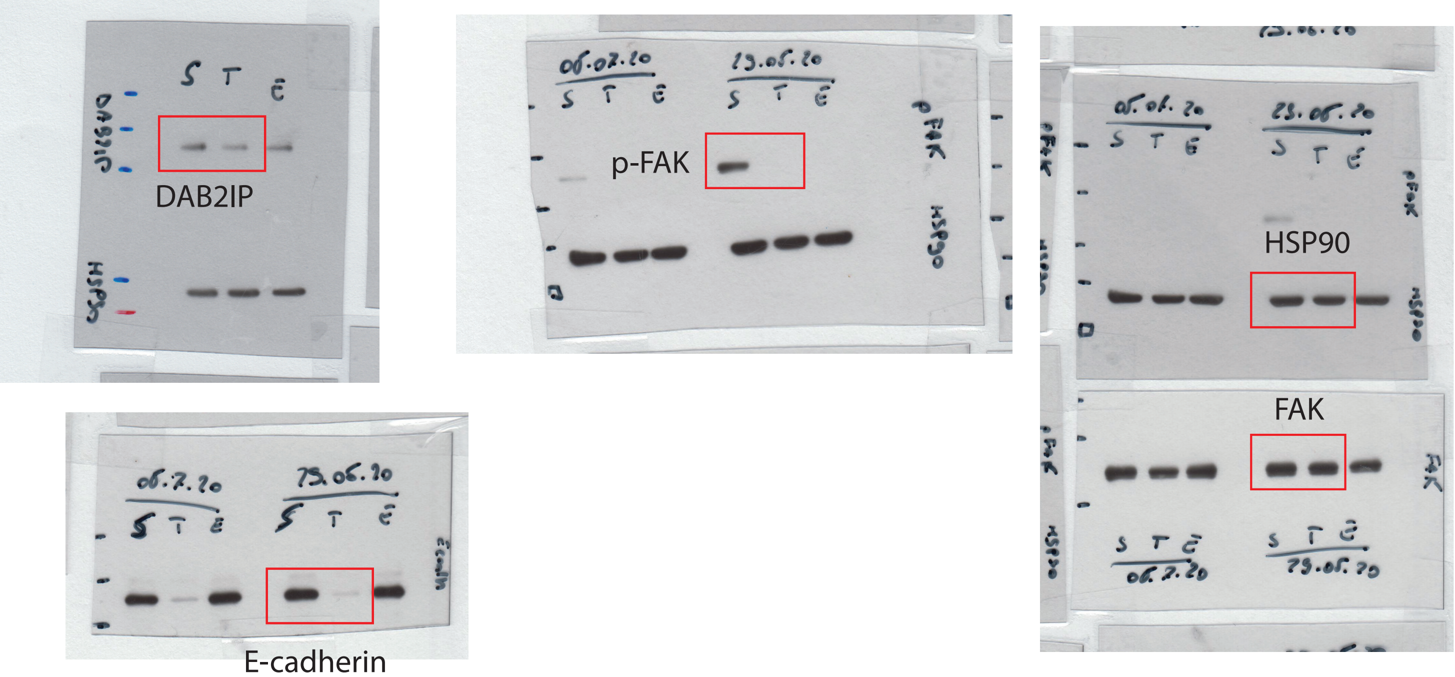

Figure 2B

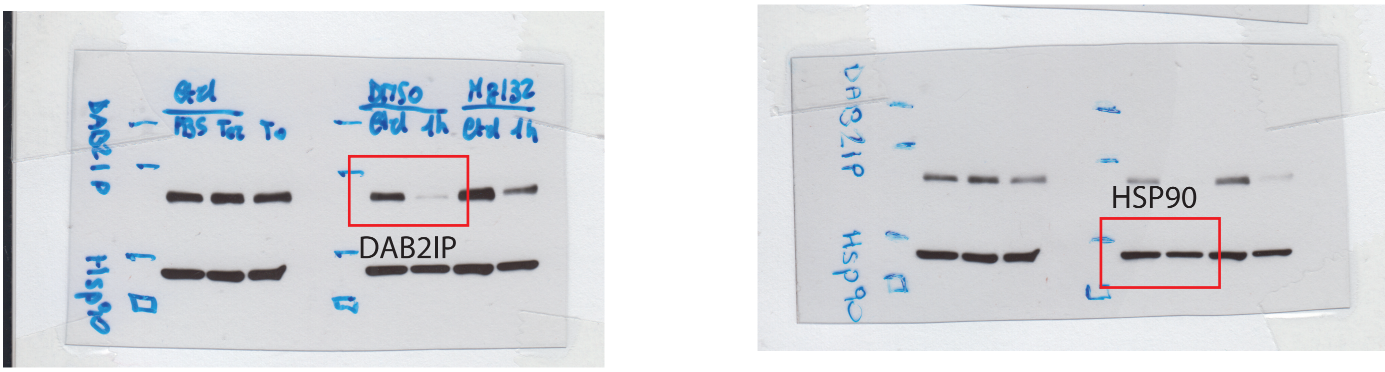

Figure 2C

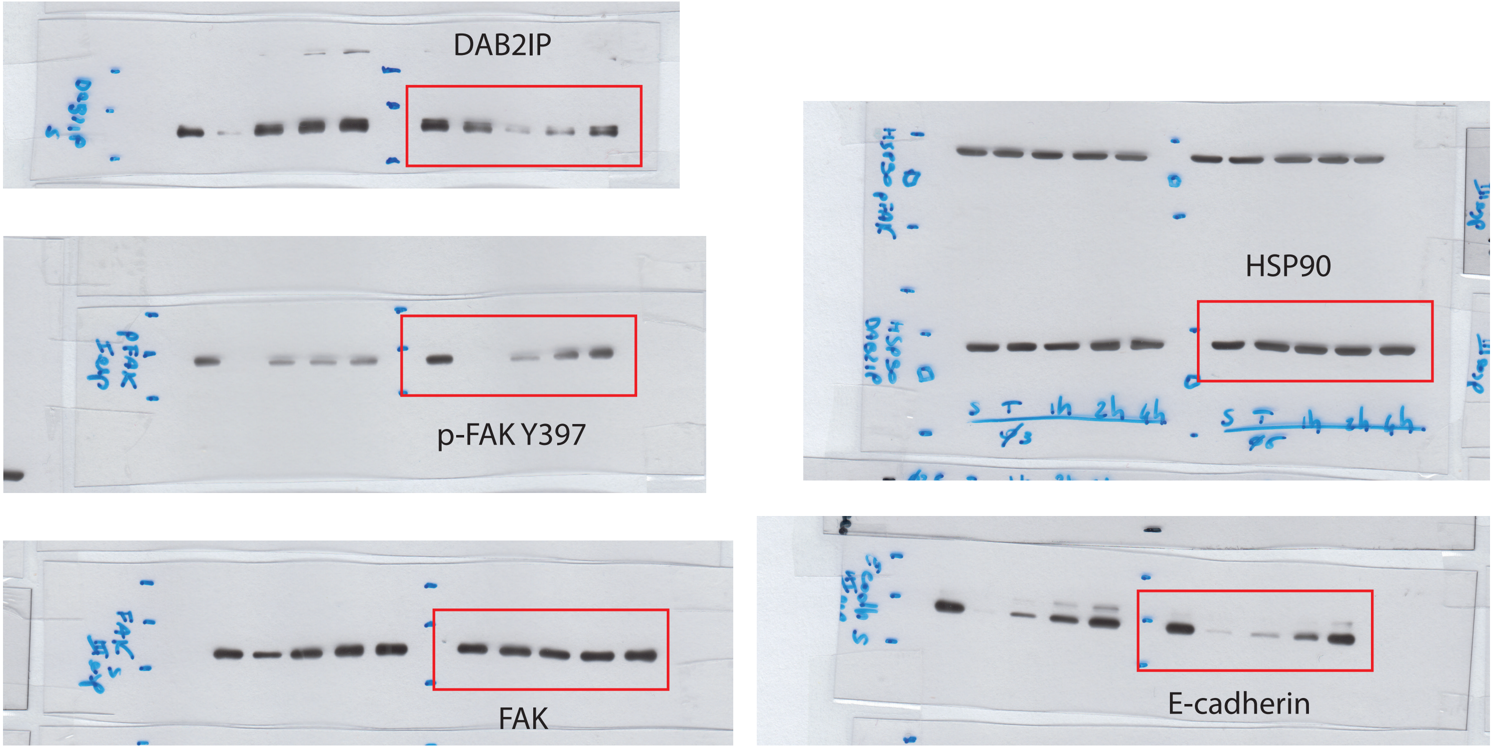

Figure 3B

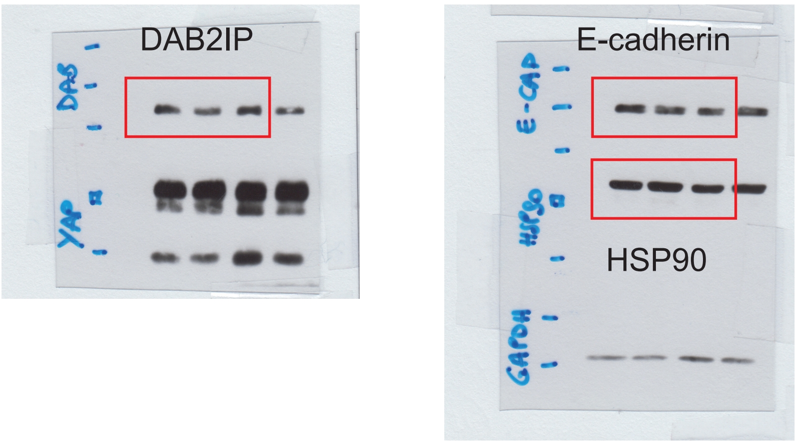

Figure 3D

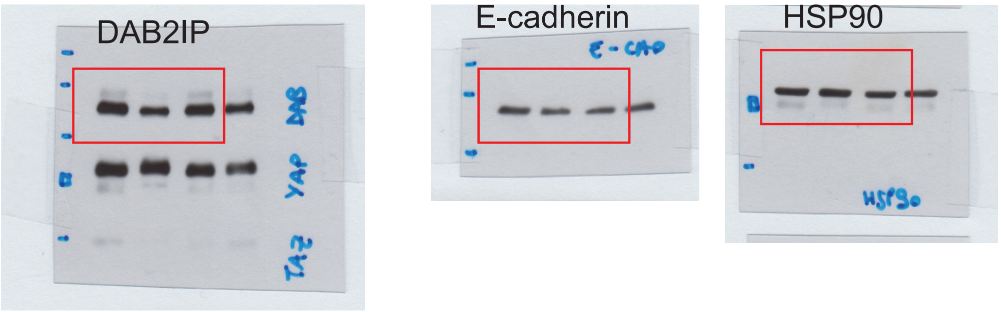

Figure 3E

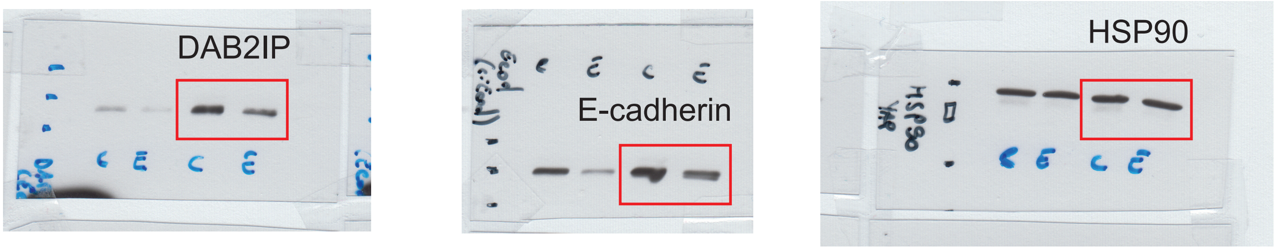

Figure 4A

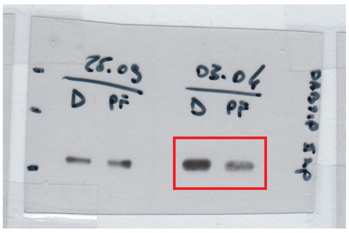

DAB2IP

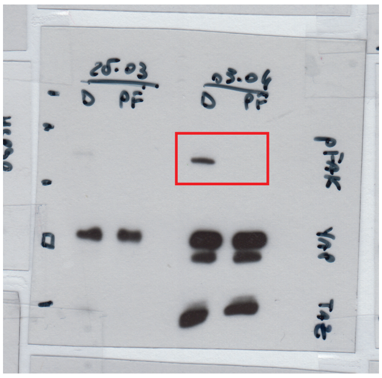

p-FAK Y397

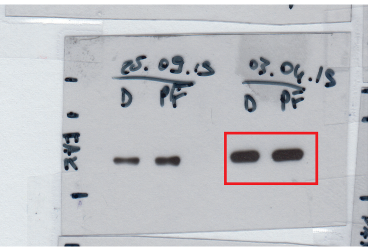

FAK

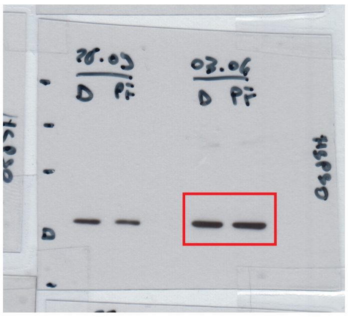

HSP90

Figure 4B

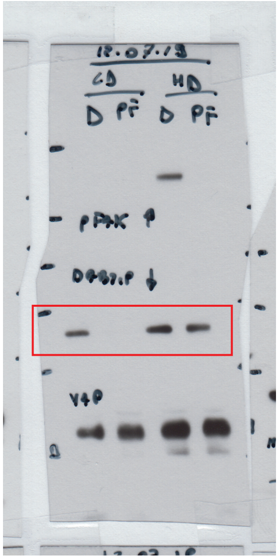

DAB2IP

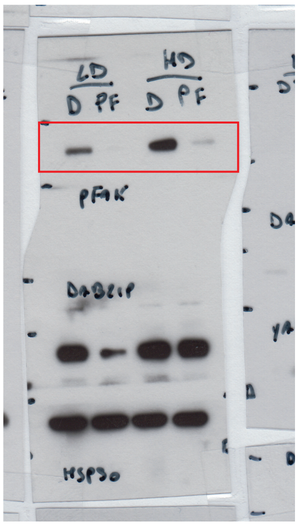

p-FAK Y397

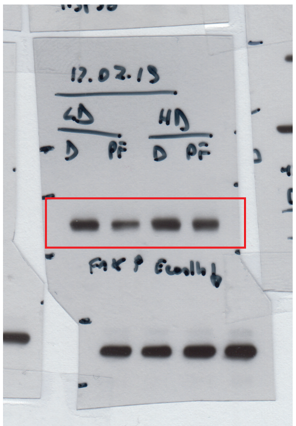

FAK

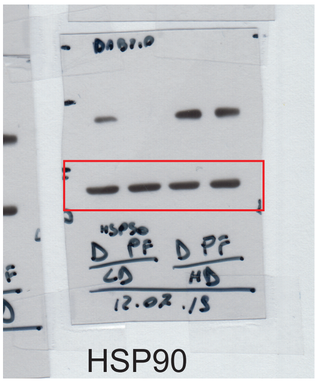

HSP90

Figure 4D: CytD

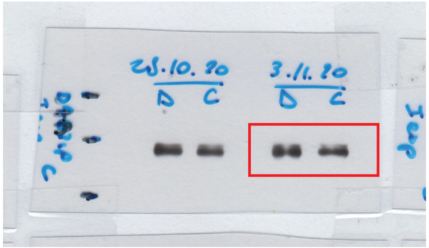

DAB2IP

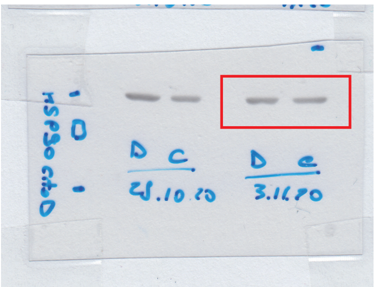

HSP90

Figure 4D: LatA

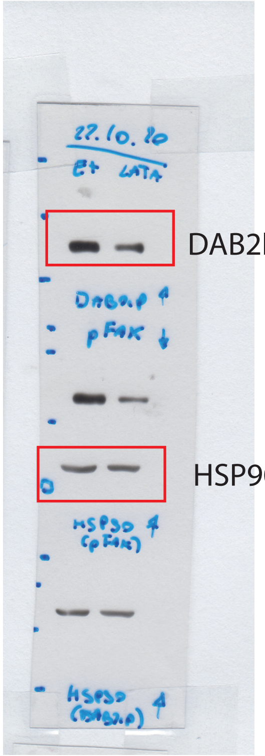

DAB2IP

HSP90

Figure 4D: Bleb

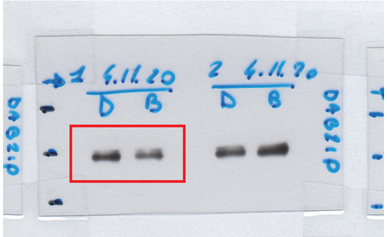

DAB2IP

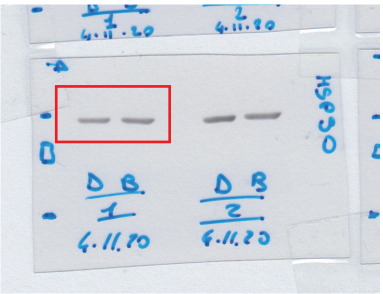

HSP90

Figure 4E

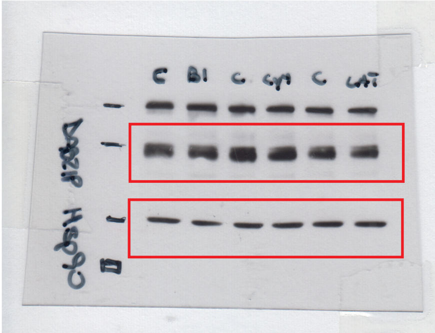

DAB2IP

HSP90

Figure 5D

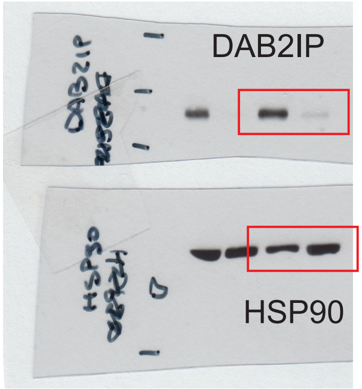

Figure 6D

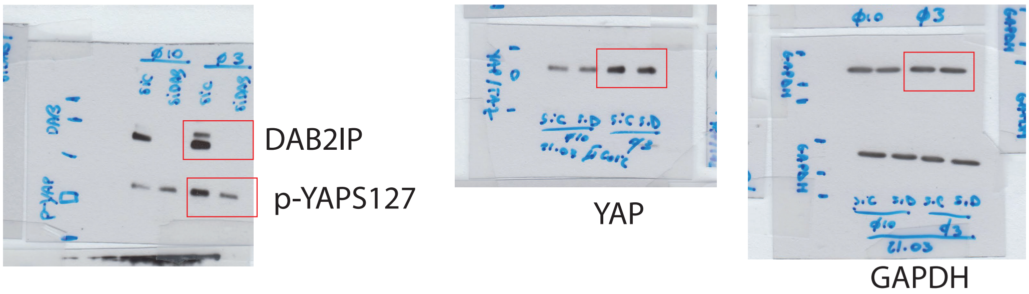

Figure 7E: left panel

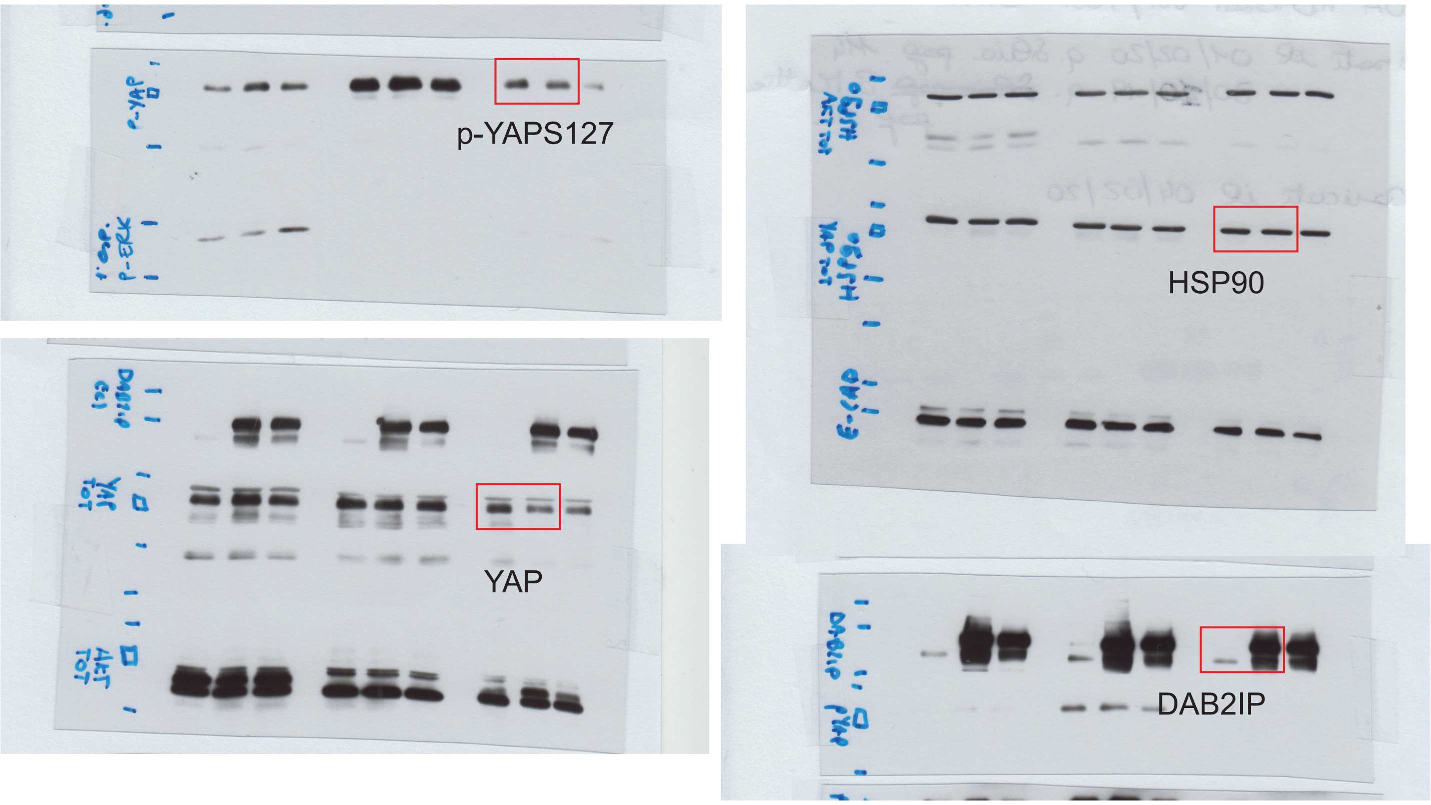

Figure 7E: right panel

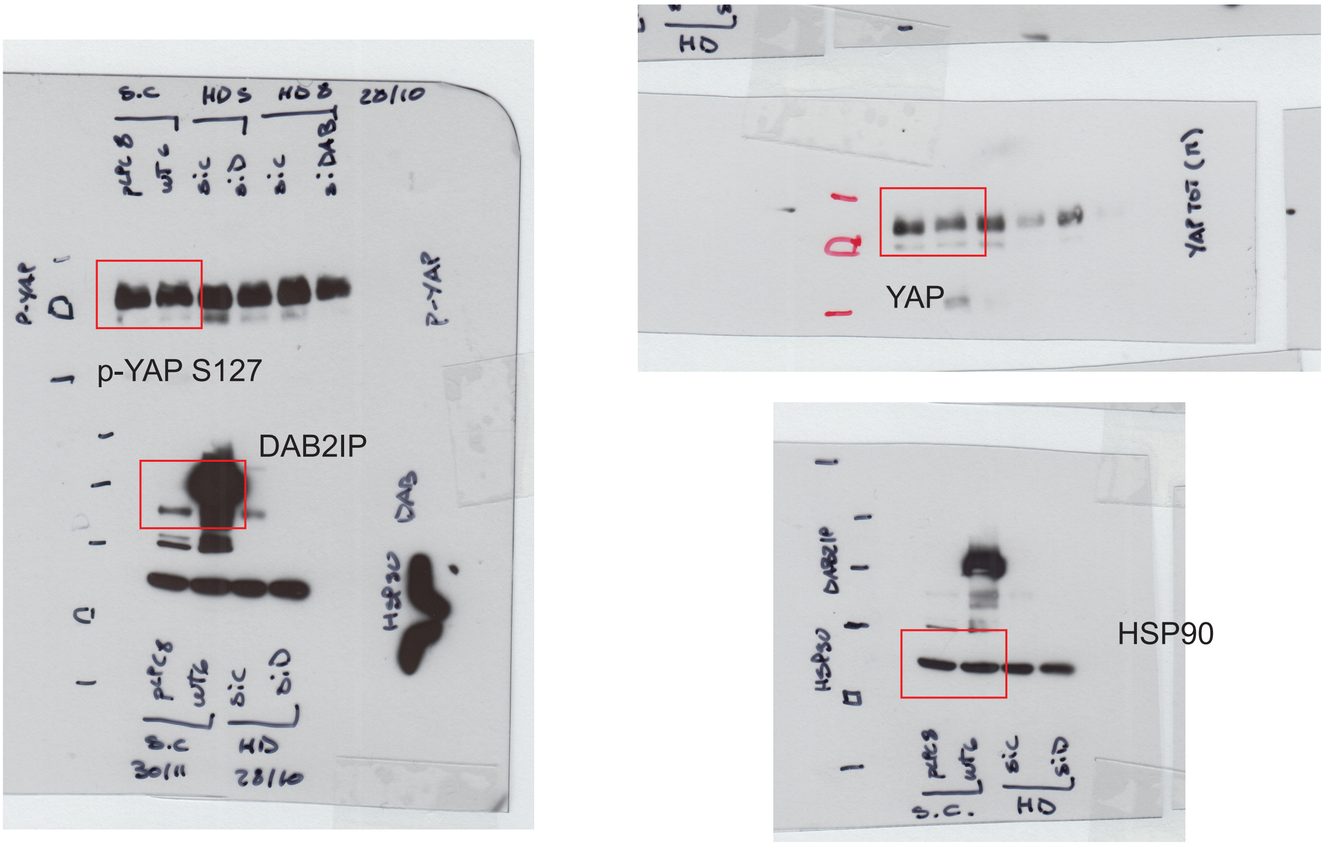

Figure 7F

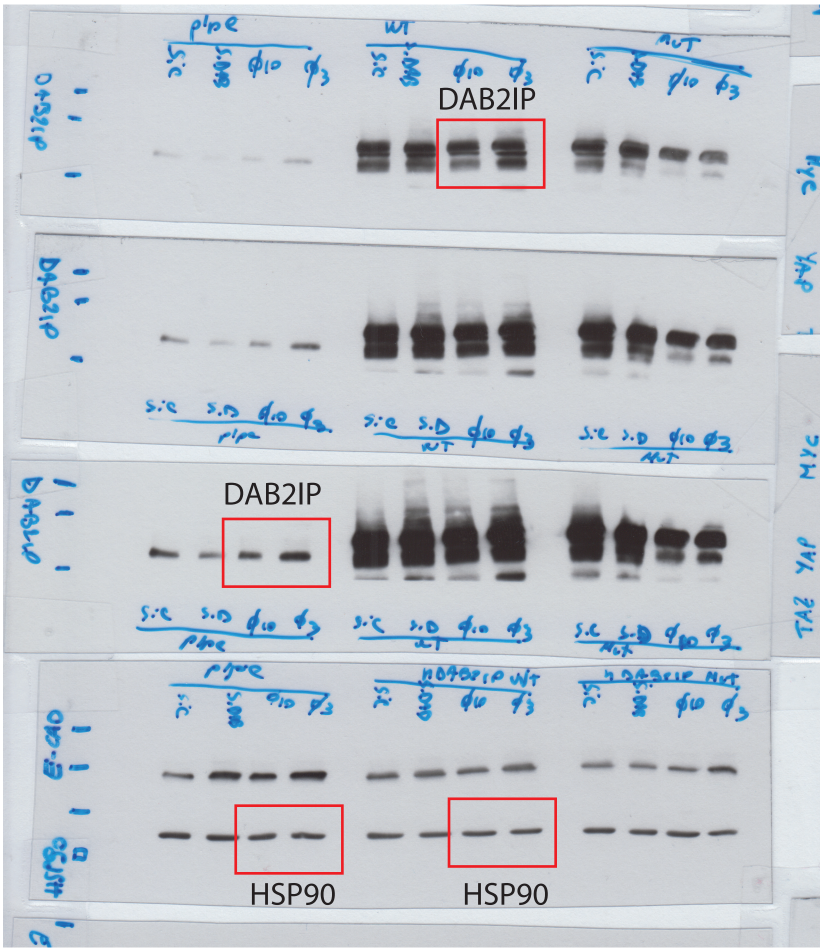

anti-DAB2IP
in hDAB2IP cl.1

anti-DAB2IP
in empty vector cl.8

anti-HSP90
in empty vector cl.8 (left)
and hDAB2IP cl.1 (right)

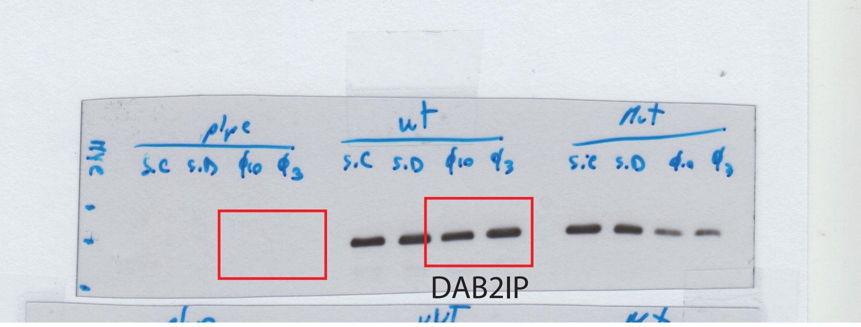

anti-Myc (9E10)
in empty vector cl.8 (left)
and hDAB2IP cl.1 (right)
