# Supplementary material for: The Tumor Suppressor DAB2IP Is Regulated by Cell Contact and Contributes to YAP/TAZ Inhibition in Confluent Cells
Source: Cancers (Basel). 2023 Jun 27;15(13):3379. doi: 10.3390/cancers15133379 (PMC10340159; doi:10.3390/cancers15133379)

Figure S1A

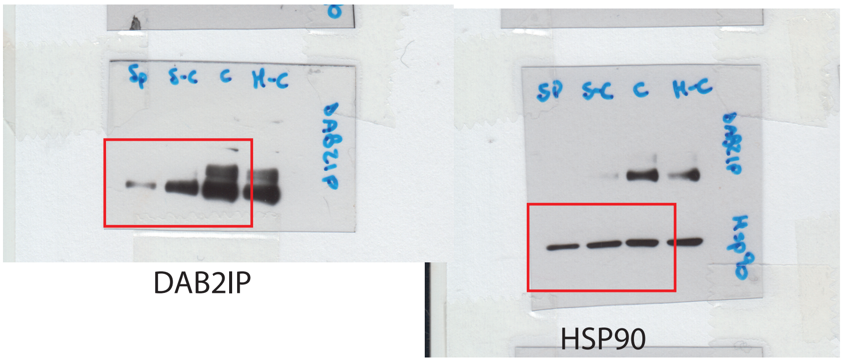

Figure S1B

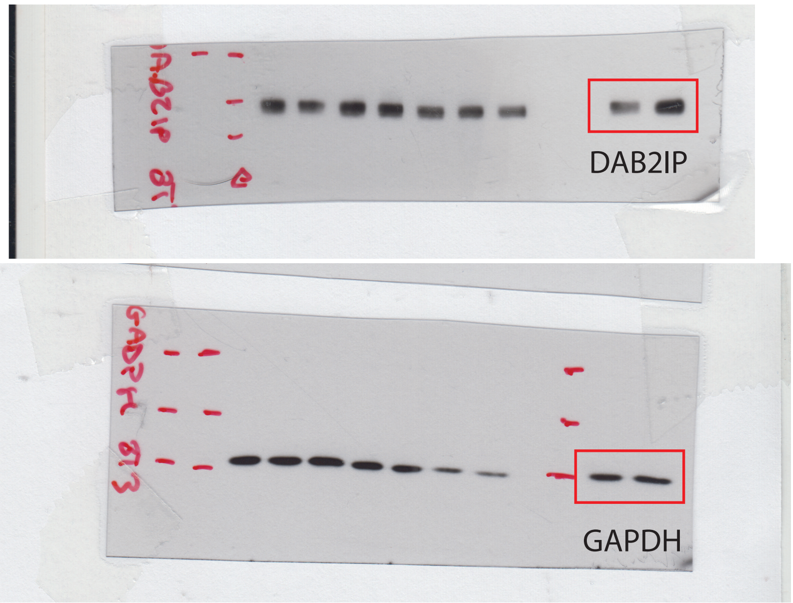

Figure S1C

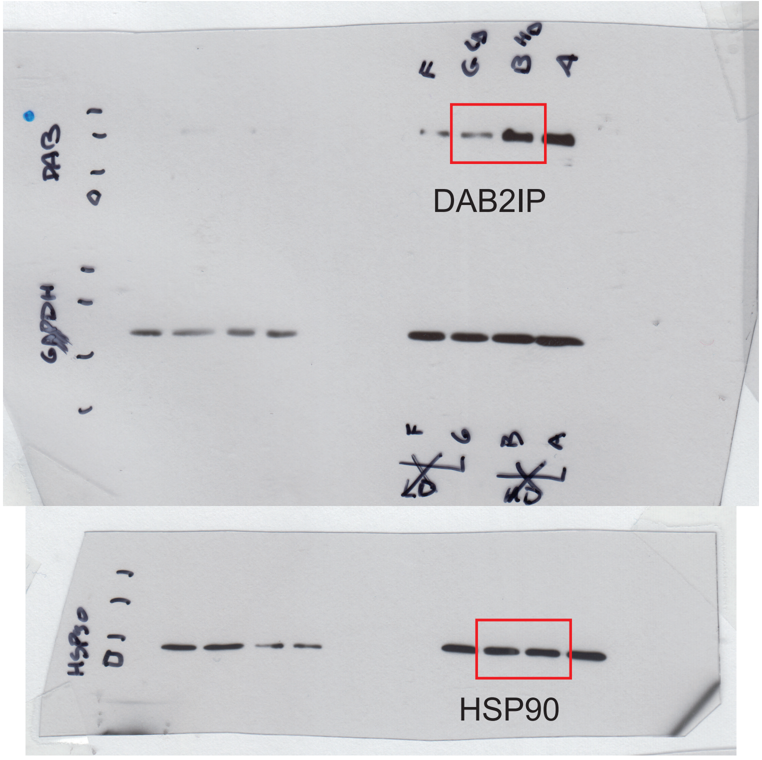

Figure S1D

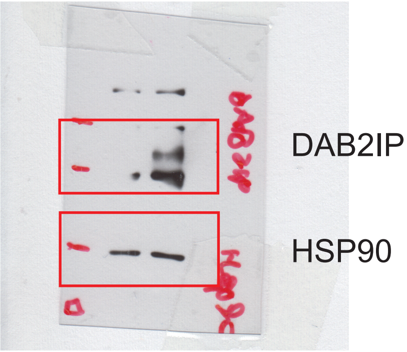

Figure S2A

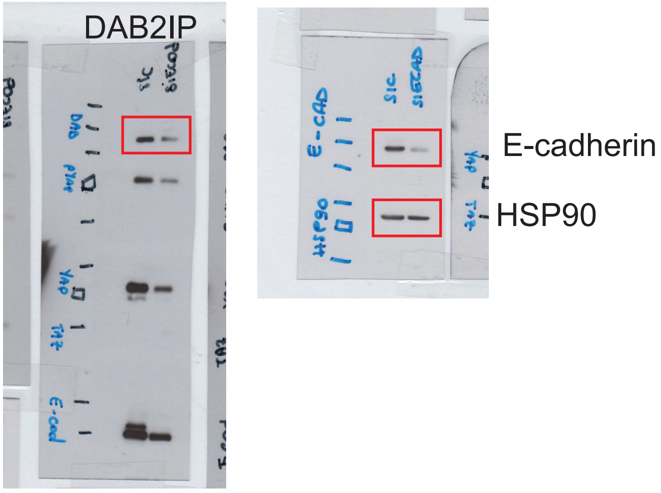

Figure S2D:

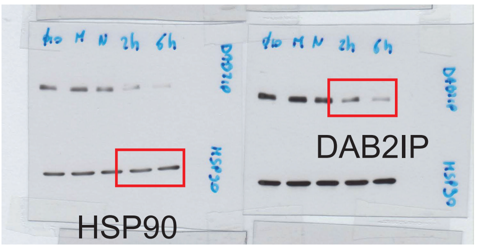

Figure S3A

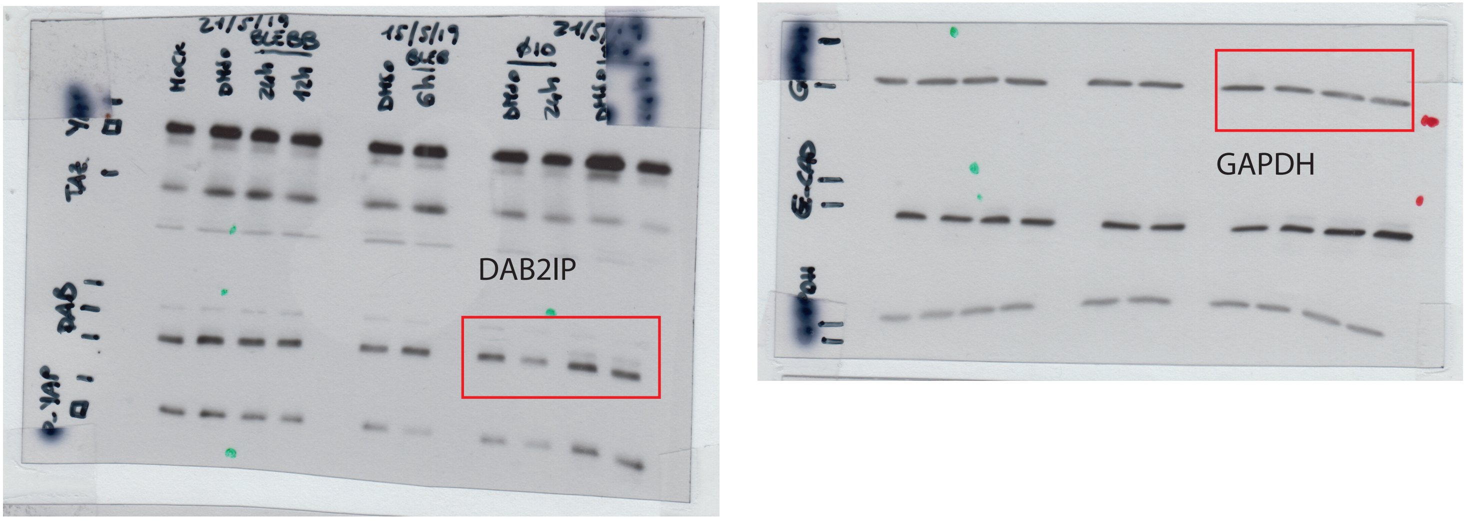

Figure S3B: subconfluent

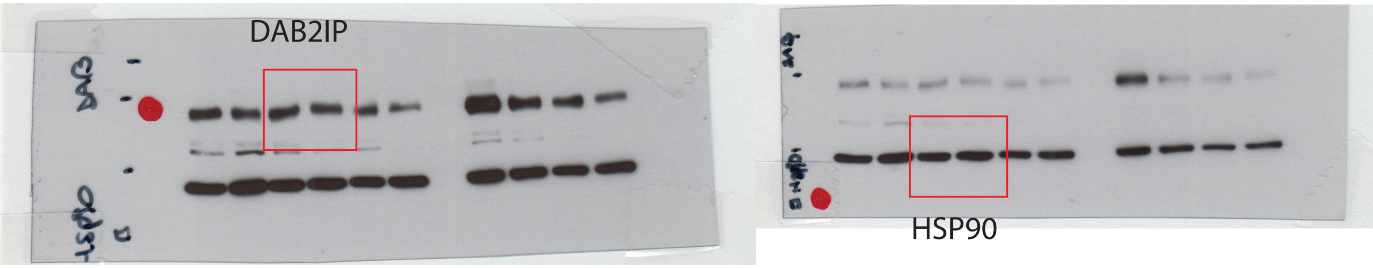

Figure S3B: confluent

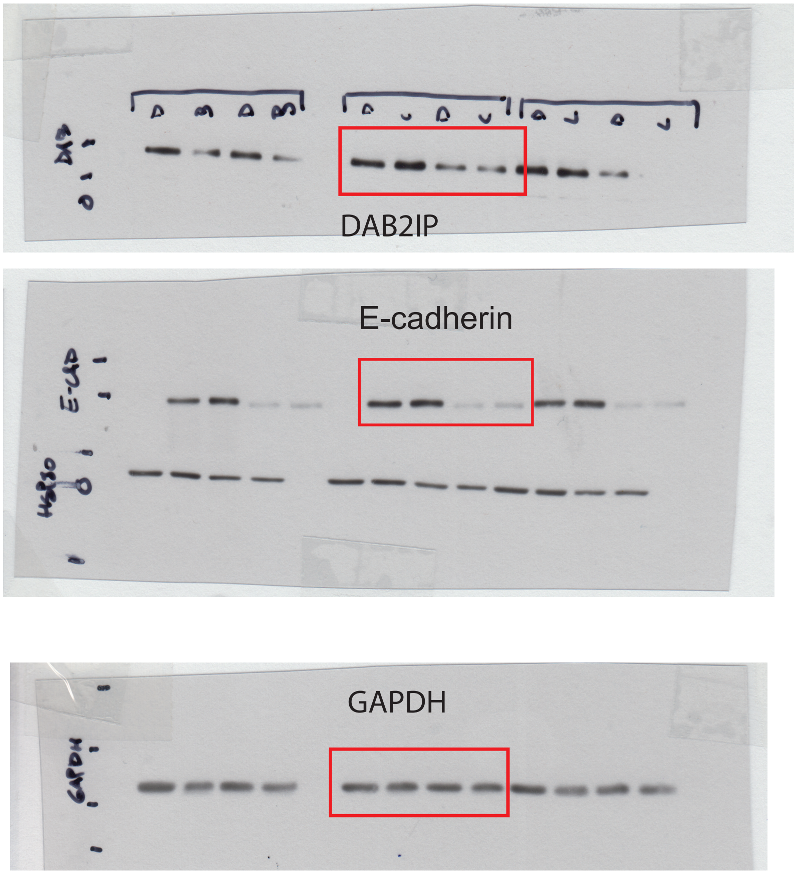

Figure S3C: subconfluent

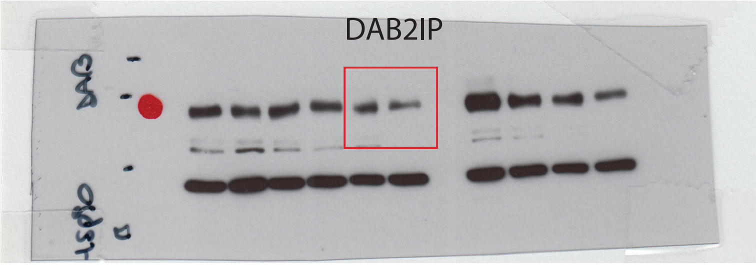

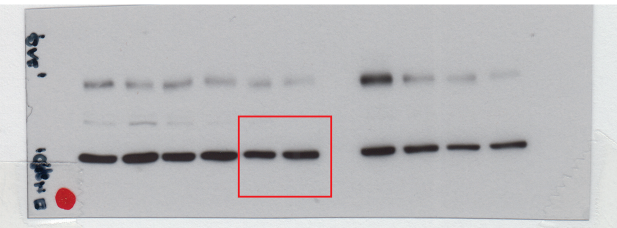

HSP90

Figure S3C: confluent

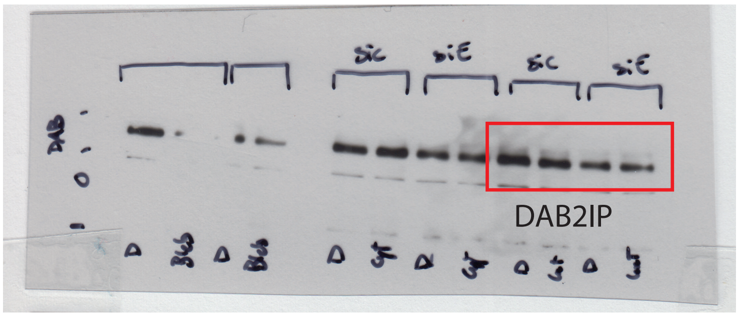

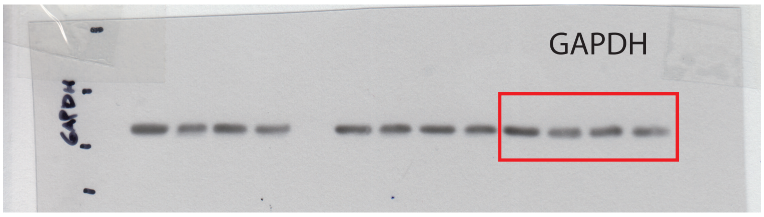

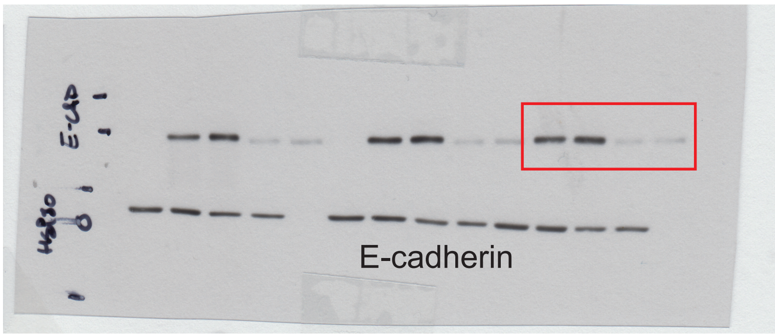

Figure S3D: LatA

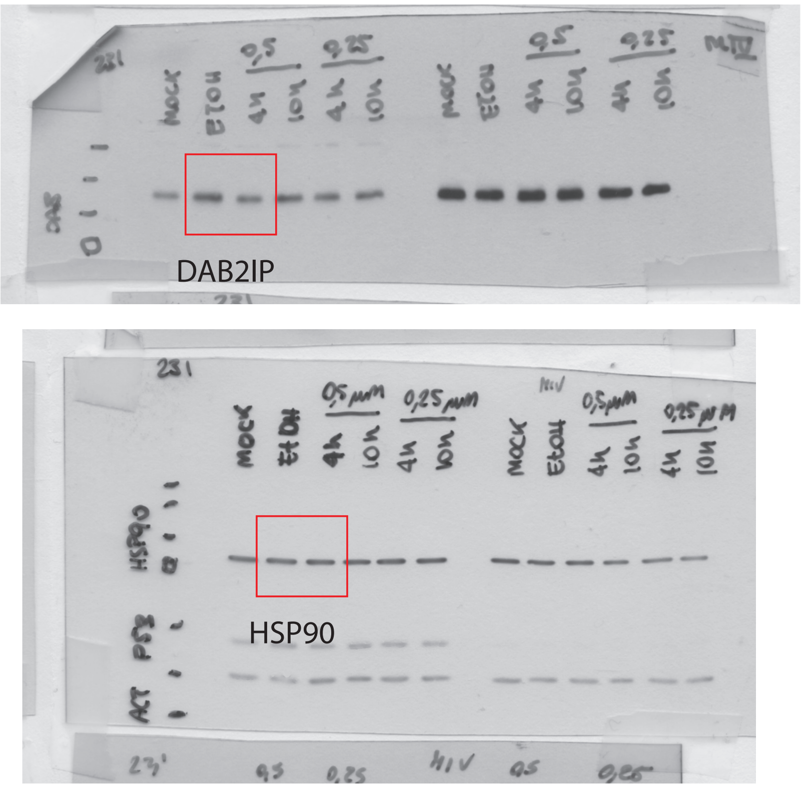

Figure S3D: CytD

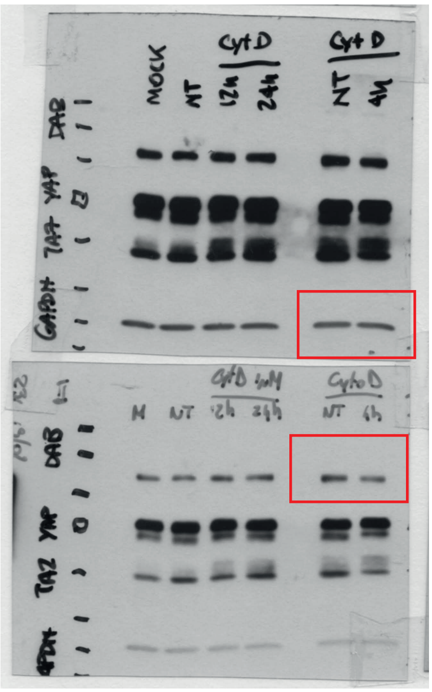

Figure S3E

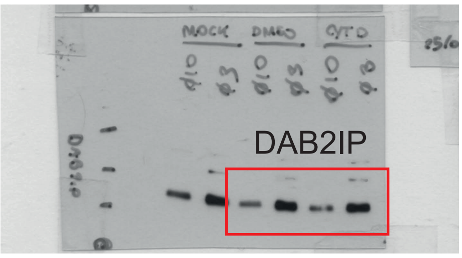

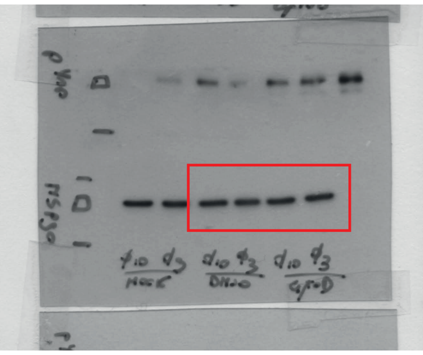

Figure S4B

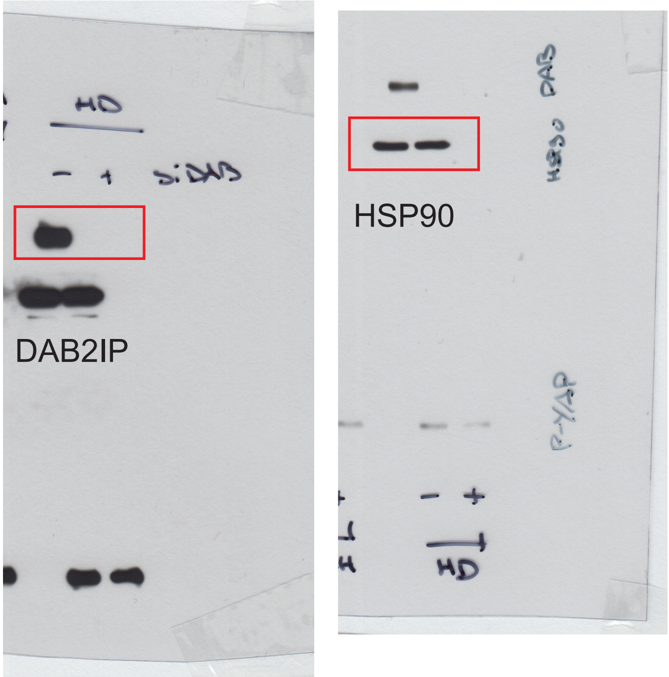

Figure S4C

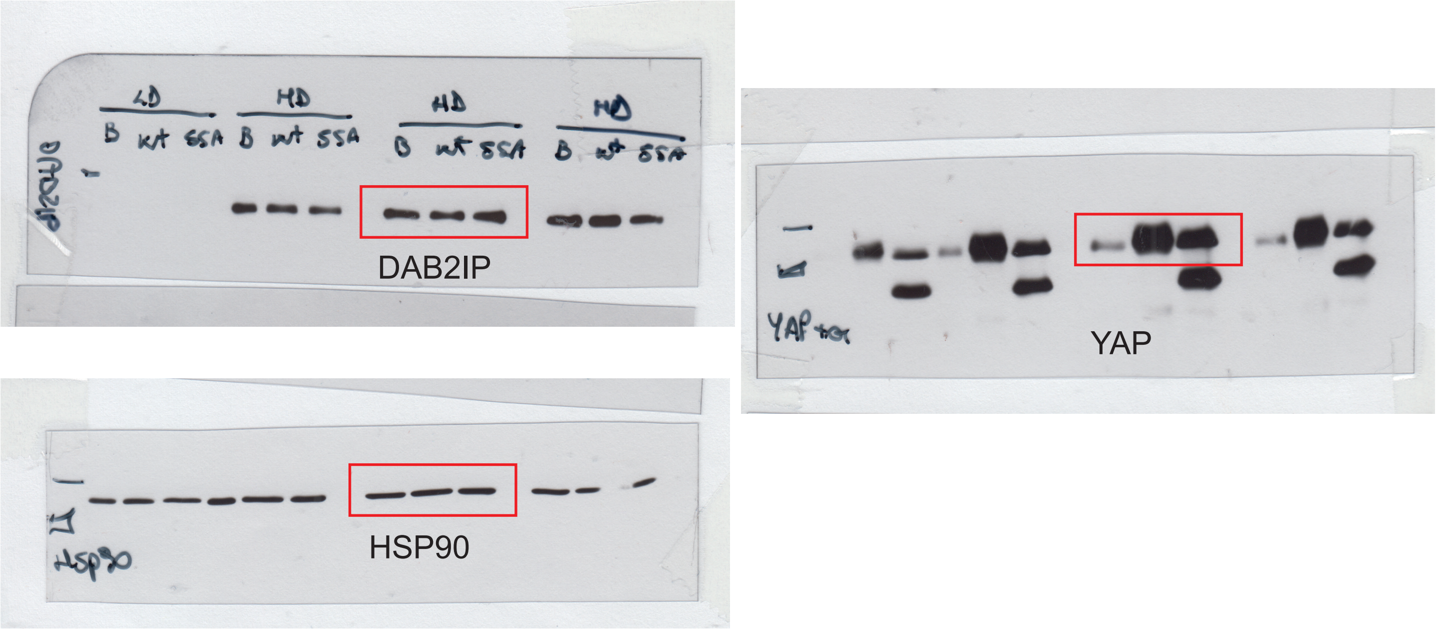

Figure S5B

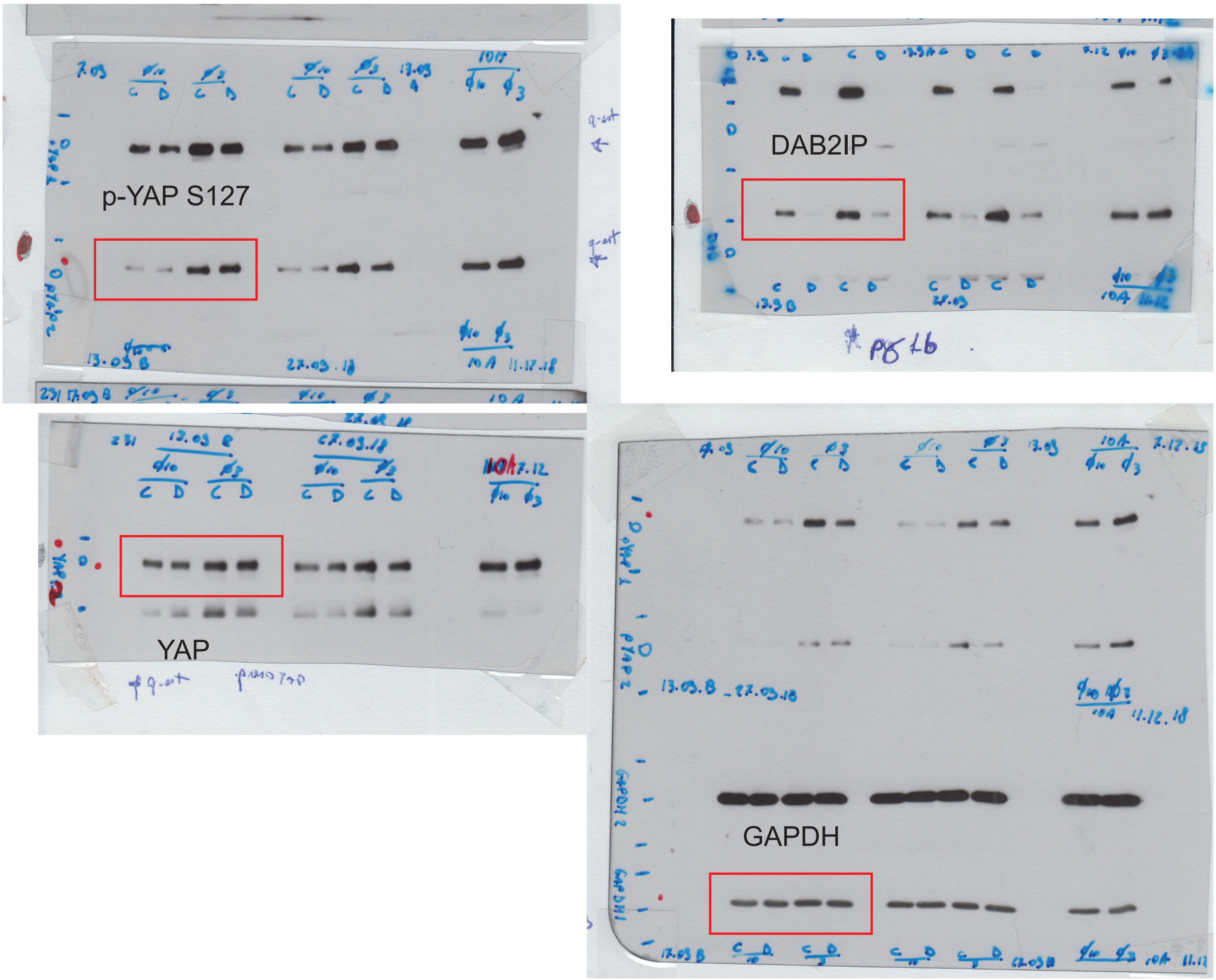

Figure S6A

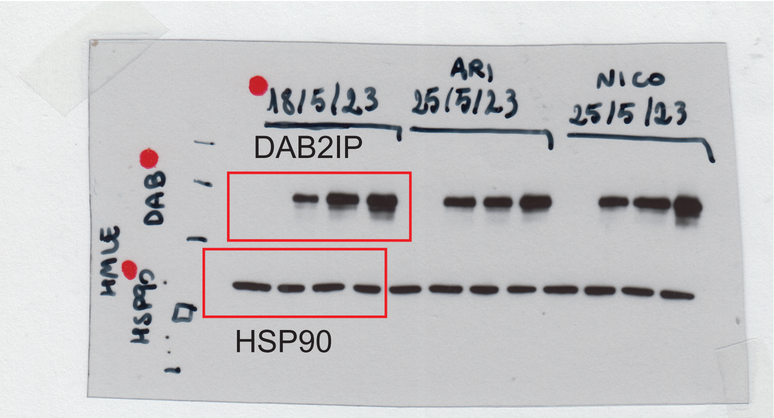

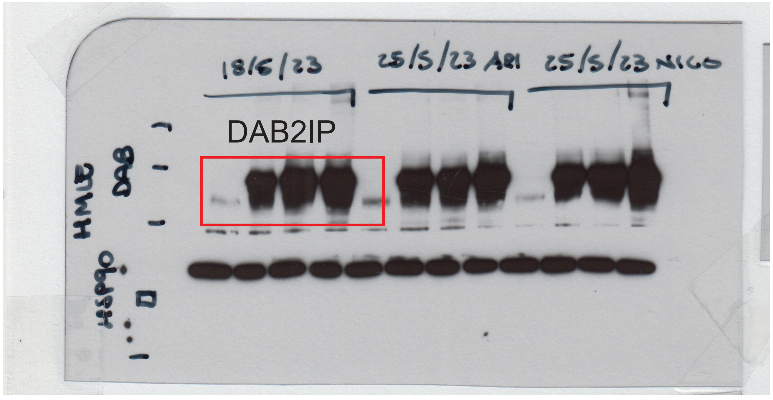

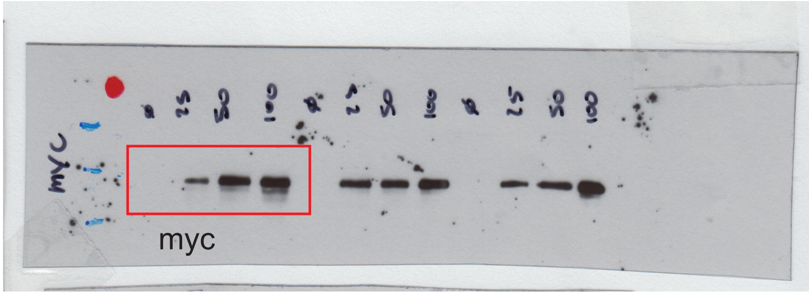

Figure S6B

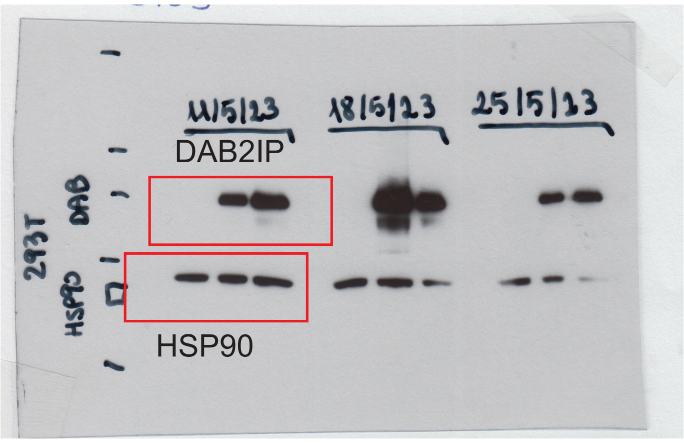

DAB2IP

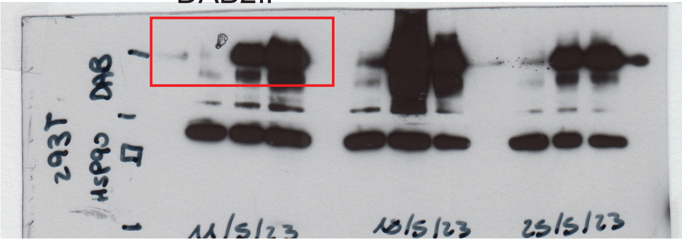

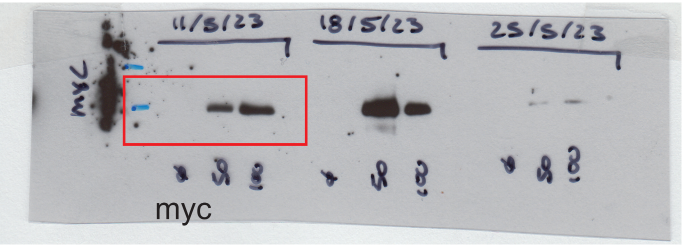

Supplement: Supplementary file 1 [file cancers-15-03379-s001.zip › File S2. Original blots Supplementary Figures Rev.pdf]
