# Supplementary material for: The Tumor Suppressor DAB2IP Is Regulated by Cell Contact and Contributes to YAP/TAZ Inhibition in Confluent Cells
Source: Cancers (Basel). 2023 Jun 27;15(13):3379. doi: 10.3390/cancers15133379 (PMC10340159; doi:10.3390/cancers15133379)
Supplement: Supplementary file 1 [file cancers-15-03379-s001.zip › Supplementary Figures S1-S6.pdf]

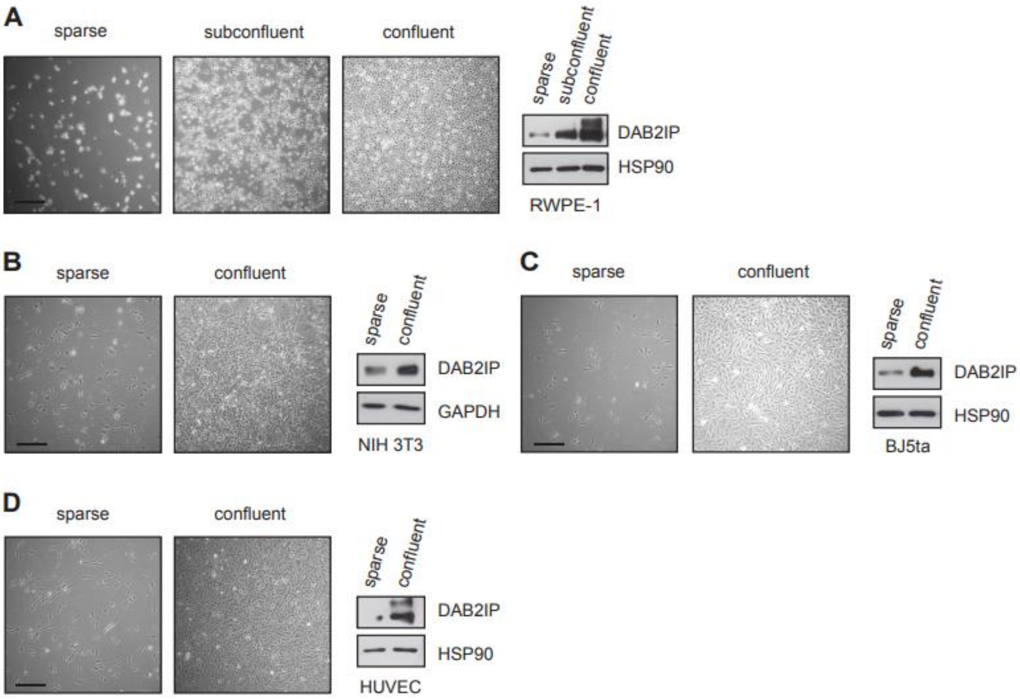

Figure S1 (related to Fig. 1). DAB2IP protein levels change with cell density in monolayer cultures.

A-D) Indicated cell lines were cultured at different densities, as detailed in Materials and Methods. After 48 hours of culture, cells were photographed, collected and cell lysate was analyzed by western blot. Representative images are shown (Scale bar = 200 μm) together with immunoblotting of DAB2IP, with HSP90 or GAPDH as loading controls.

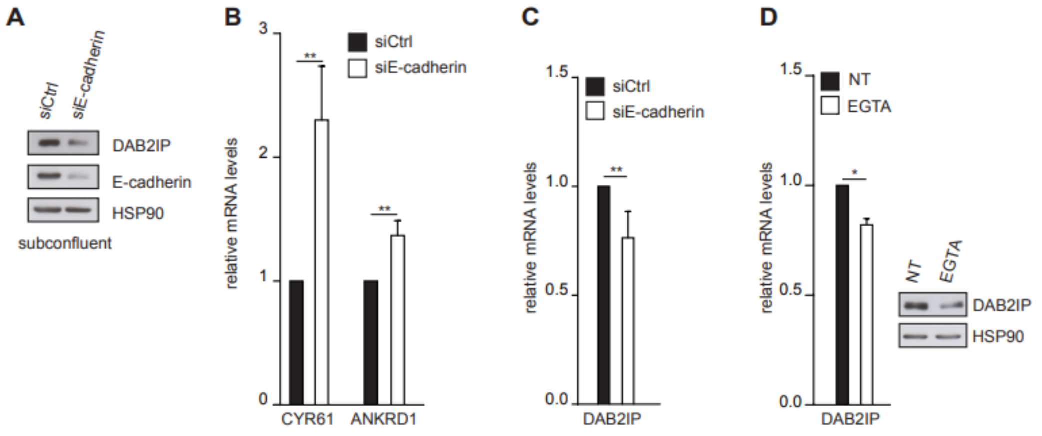

Figure S2 (related to Fig. 3). Cell-cell interactions affect DAB2IP expression.

A-C) E-cadherin depletion downregulates DAB2IP and stimulates YAP/TAZ activity. A) MCF10A cells were transfected with the indicated siRNAs and cultured for 48 hours until sub-confluent. DAB2IP and E-cadherin levels were detected by immunoblotting, with HSP90 as a loading control. B) Expression of CYR61 and ANKRD1 was measured by RT-qPCR. Data are normalized on histone H3 (mean ± SD; n =3; ** P<0.01). C) Expression of DAB2IP was measured by RT-qPCR. Data are normalized on histone H3 (mean ± SD; n = 4; ** P<0.01) C).

D) EGTA treatment downregulates DAB2IP. MCF10A cells were cultured for 48 hours after seeding at confluence (60000 cells/cm2). Confluent cells were then exposed to 10 mM EGTA for 3 hours. Expression of DAB2IP was measured by RT-qPCR. Data are normalized on histone H3 (mean ± SD; n =3; *P<0.05). DAB2IP protein levels were detected by immunoblotting, with HSP90 as a loading control.

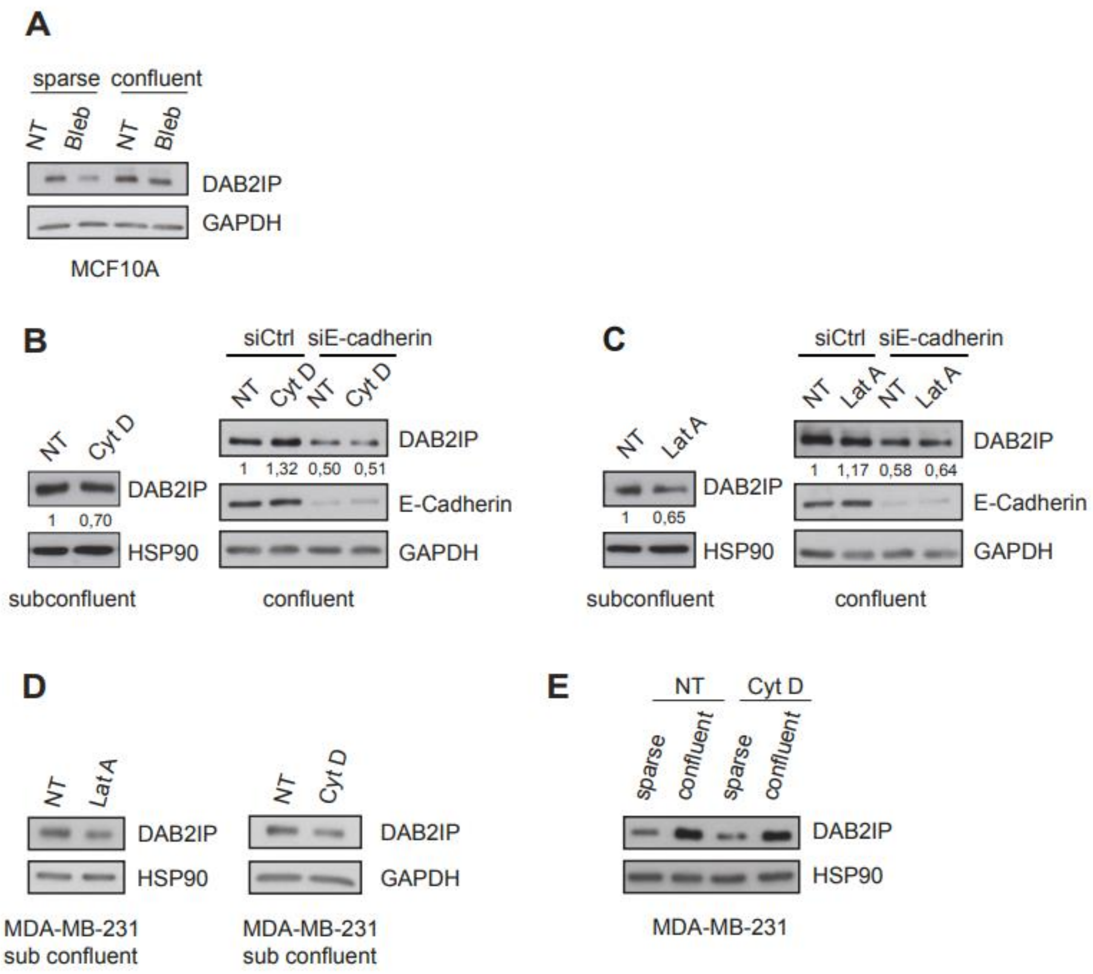

Figure S3 (related to Fig. 4). Cytoskeletal tension is not necessary to sustain DAB2IP protein levels in confluent cells.

A) Blebbistatin decreases DAB2IP in sparse but not confluent cells. MCF10A were seeded at low (2500 cells/cm2) or high density (60000 cells/cm2), cultured to sparse or confluent conditions, then exposed to 50 µM Blebbistatin (Bleb) for 24 hours before lysis. DAB2IP was detected by immunoblotting, with HSP90 as loading control.

B-C) Effects of Cytochalasin D and Latrunculin A in combination with e-cadherin depletion. MCF10A cells were transfected with the indicated siRNAs for 24 hours, then seeded and cultured for 48 hours to subconfluent or confluent condition. Cells were treated with 10 µM Cytochalasin D (Cyt D) or 0.5 µM Latrunculin A (LatA) for 4 hours before lysis. DAB2IP and e-cadherin were detected by immunoblotting, with HSP90 or GAPDH as loading controls. Bands were quantified by densitometry of autoradiography film, and normalized to Hsp90/GAPDH.

D-E) Effects of Latrunculin A and Cytochalasin D on DAB2IP protein levels in MDA-MB-231 cancer cells. B) MDA-MB-231 cells were cultured to subconfluent condition, then exposed to 0.5 µM Latrunculin A (LatA) or 10 µM Cytochalasin D (Cyt D) for 4 hours. DAB2IP was detected by immunoblotting, with HSP90 or GAPDH as loading controls. C) MDA-MB-231 cells were seeded at low (5000 cells/cm2) or high density (70000 cells/cm2), cultured to sparse or confluent conditions, then exposed to 10 µM Cytochalasin D (Cyt D) for 4 hours. DAB2IP was detected by immunoblotting, with HSP90 as loading control.

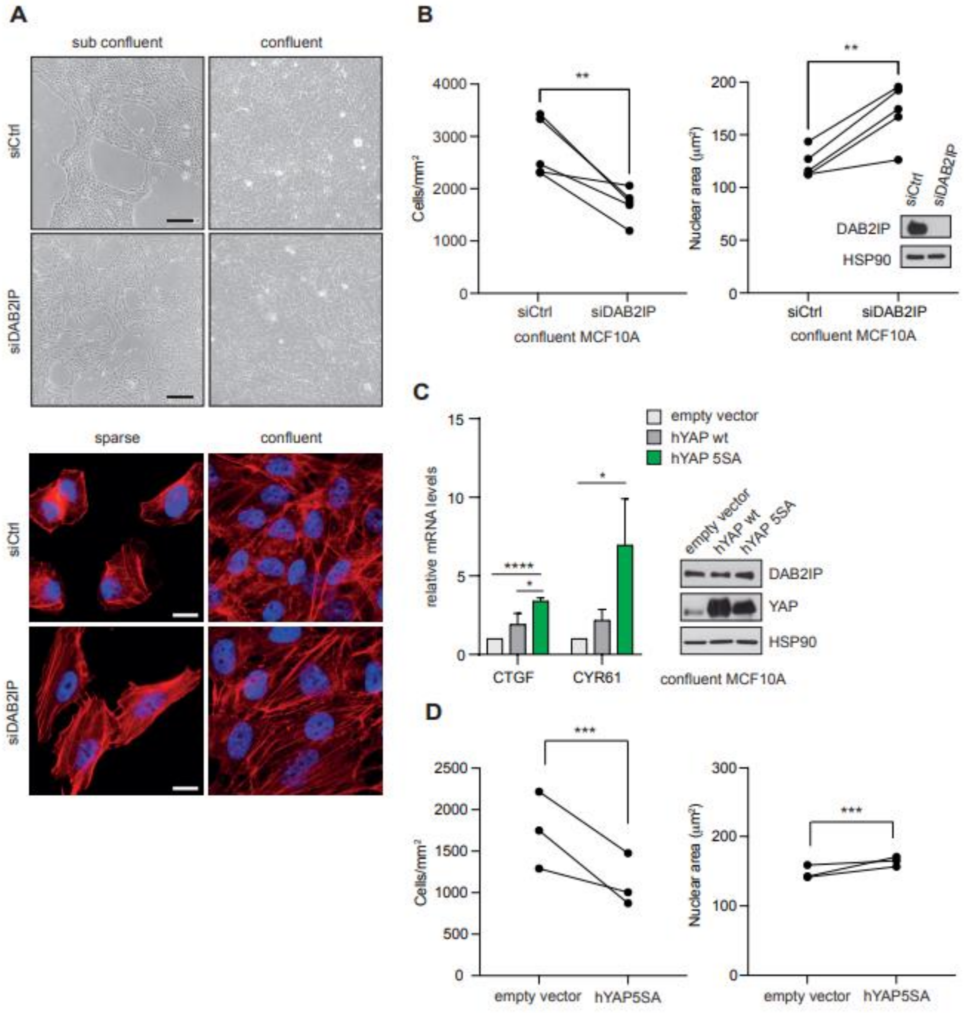

Figure S4 (related to Fig. 5). DAB2IP depletion or YAP overexpression modify the morphology of confluent cells.

A) DAB2IP depletion affects the shape of mammary epithelial cells. MCF10A cells were transfected with the indicated siRNAs (40nM) for 24 hours, then seeded and cultured for 48 hours to a sparse, subconfluent, or confluent condition. Representative brightfield (scale bar = 200 µm) and fluorescence (red = phalloidin; blue = Hoechst; scale bar = 10 µm) images of cells at the indicated densities are shown.

B) DAB2IP depletion reduces maximal cell density and increases nuclear size in a confluent monolayer. Left graph represents the average number of nuclei per area counted in Figure 5A, measured in five independent experiments (** $P<0.01$). Right graph represents the average nuclear area of the cells counted in Figure 5C, measured in five independent experiments (** $P<0.01$). Inset shows an immunoblotting control of DAB2IP knockdown efficiency.

C) Characterization of mammary epithelial cells stably expressing YAP constructs. MCF10A cells stably overexpressing wild-type hYAP, or constitutively active hYAP 5SA, or an empty vector control, were seeded at high density and cultured for 48h to a confluent monolayer. Expression of YAP/TAZ target genes was measured by RT-qPCR; data were normalized on H3 (mean ± SD; n =3). Immunoblotting on the right confirmed overexpression of ectopic YAP proteins.

D) YAP overexpression reduces maximal cell density and increases nuclear size in a confluent monolayer. Left graph represents the average number of nuclei per area counted in Figure 5E, measured in three independent experiments (* $P<0.05$, *** $P<0.001$). Right graph represents the average nuclear area of the cells counted in Figure 5F, measured in three independent experiments (** $P<0.01$, *** $P<0.001$).

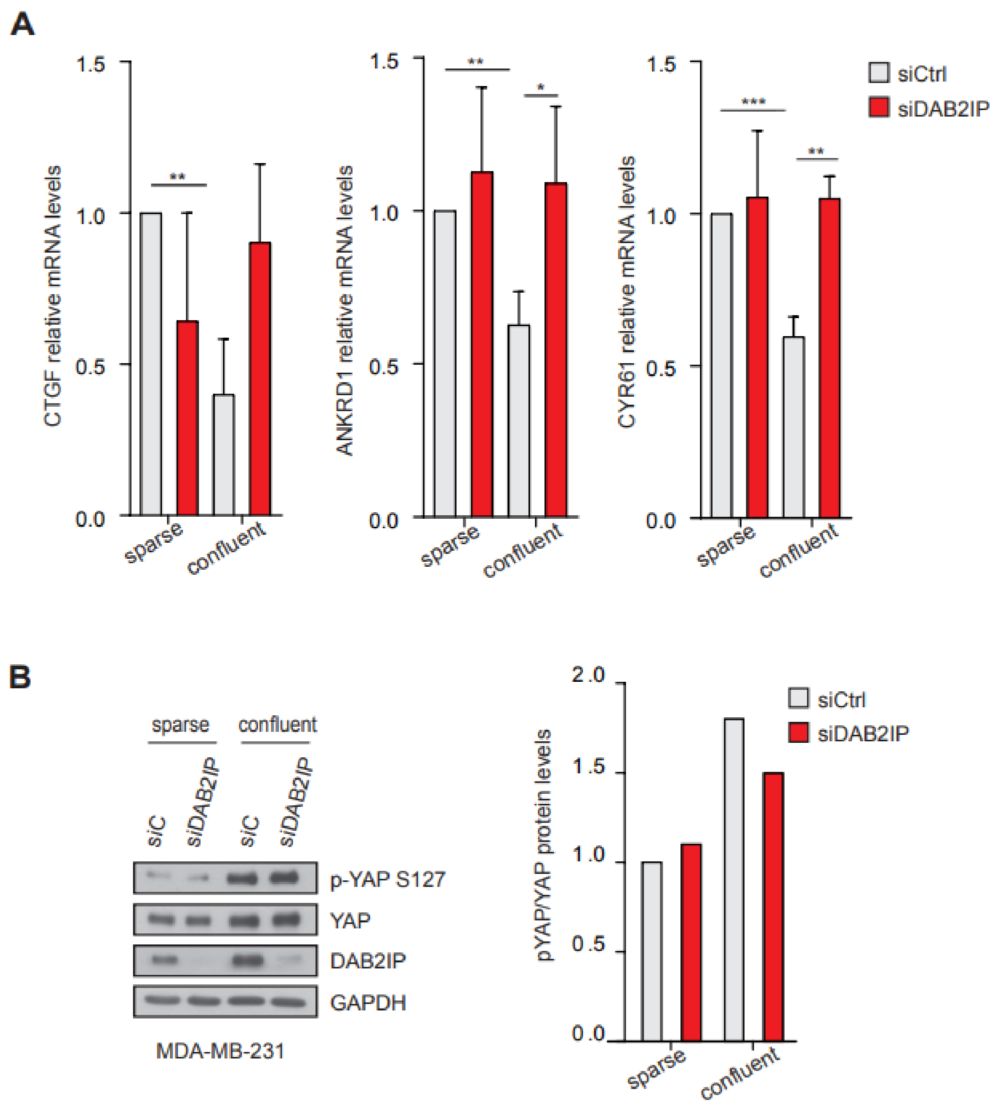

Figure S5 (related to Fig. 6). DAB2IP depletion allows YAP/TAZ activation in breast cancer MDA-MB-231 cells.

A) DAB2IP depletion increases expression of YAP/TAZ target genes in MDA-MB-231. Cells were transfected with the indicated siRNAs (40nM) for 24 hours, then seeded at high density (70000 cells/cm2) and cultured for additional 48 hours, until confluency. Expression of CTGF, ANKRD1 and CYR61 was measured by RT-qPCR. Data were normalized on histone H3 (mean ± SD; n =3; *P<0.05, ***P<0.001, ****P<0.0001).

B) DAB2IP depletion reduces YAP Ser127 phosphorylation in confluent MDA-MB-231. Cells were cultured as in A. DAB2IP, phosphorylated (p-S127) and total YAP were detected by immunoblotting, with GAPDH as loading control. Protein bands were quantified and normalized to GAPDH by densitometry of autoradiography film (histogram on the right).

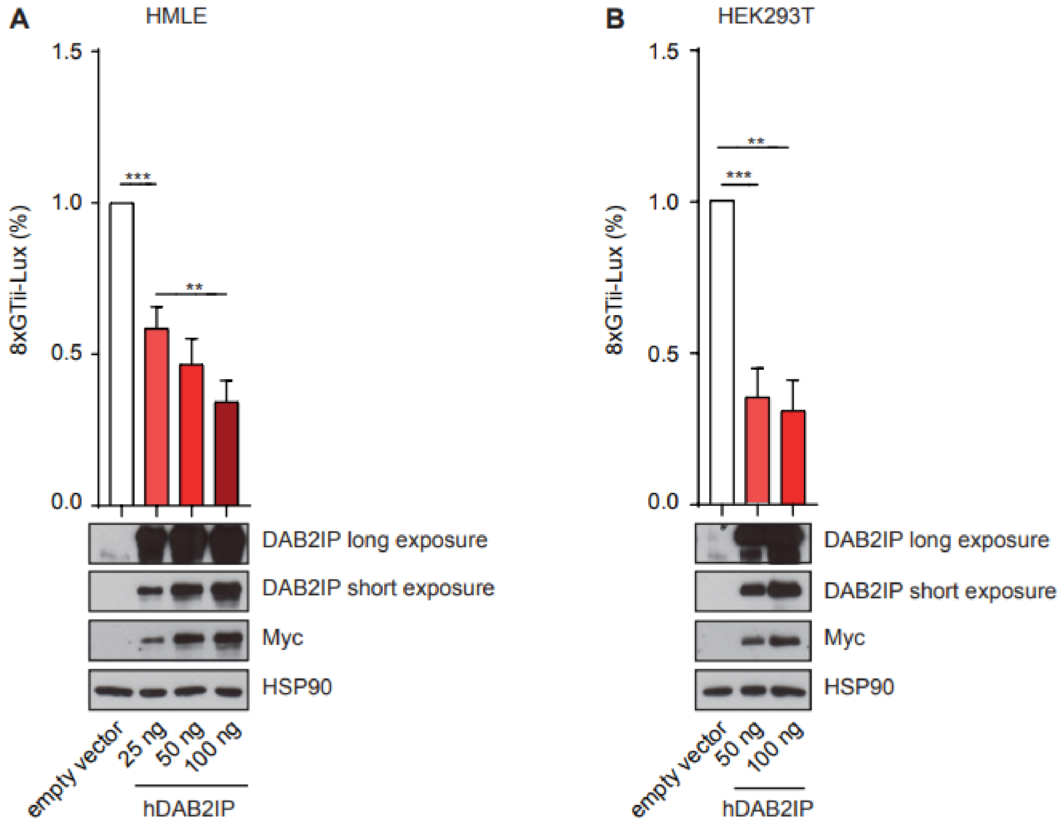

Figure S6 (related to Fig. 7). DAB2IP overexpression inhibits YAP/TAZ activity.
The indicated cells growing in sub-confluent conditions were transfected with a fixed amount of the 8xGTIIC-Lux reporter together with a small amount of pCMV-Renilla plasmid, and increasing amounts of Myc-hDAB2IP expression plasmid. After 24 hours YAP/TAZ activity was measured by Dual Luciferase assay (mean ± SD; n=3; **P<0.01, ***P<0.001).
